# Ketogenic diets slow melanoma growth in vivo regardless of tumor genetics and metabolic plasticity

**DOI:** 10.1186/s40170-022-00288-7

**Published:** 2022-07-18

**Authors:** Daniela D. Weber, Sepideh Aminzadeh-Gohari, Maheshwor Thapa, Anna-Sophia Redtenbacher, Luca Catalano, Tânia Capelôa, Thibaut Vazeille, Michael Emberger, Thomas K. Felder, René G. Feichtinger, Peter Koelblinger, Guido Dallmann, Pierre Sonveaux, Roland Lang, Barbara Kofler

**Affiliations:** 1 Research Program for Receptor Biochemistry and Tumor Metabolism, Department of Pediatrics, University Hospital of the Paracelsus Medical University, 5020 Salzburg, Austria; 2 Biocrates Life Sciences AG, 6020 Innsbruck, Austria; 3 Institut de Recherche Expérimentale et Clinique (IREC), Université catholique de Louvain (UCLouvain), 1200 Brussels, Belgium; 4 Patholab Salzburg, 5020 Salzburg, Austria; 5 Department of Laboratory Medicine, University Hospital of the Paracelsus Medical University, 5020 Salzburg, Austria; 6 Department of Dermatology and Allergology, University Hospital of the Paracelsus Medical University, 5020 Salzburg, Austria

**Keywords:** Ketogenic diet, Melanoma, Cancer metabolism, Metabolomics

## Abstract

**Background:**

Growing evidence supports the use of low-carbohydrate/high-fat ketogenic diets as an adjunctive cancer therapy. However, it is unclear which genetic, metabolic, or immunological factors contribute to the beneficial effect of ketogenic diets. Therefore, we investigated the effect of ketogenic diets on the progression and metabolism of genetically and metabolically heterogeneous melanoma xenografts, as well as on the development of melanoma metastases in mice with a functional immune system.

**Methods:**

Mice bearing BRAF mutant, NRAS mutant, and wild-type melanoma xenografts as well as mice bearing highly metastatic melanoma allografts were fed with a control diet or ketogenic diets, differing in their triglyceride composition, to evaluate the effect of ketogenic diets on tumor growth and metastasis. We performed an in-depth targeted metabolomics analysis in plasma and xenografts to elucidate potential antitumor mechanisms in vivo.

**Results:**

We show that ketogenic diets effectively reduced tumor growth in immunocompromised mice bearing genetically and metabolically heterogeneous human melanoma xenografts. Furthermore, the ketogenic diets exerted a metastasis-reducing effect in the immunocompetent syngeneic melanoma mouse model. Targeted analysis of plasma and tumor metabolomes revealed that ketogenic diets induced distinct changes in amino acid metabolism. Interestingly, ketogenic diets reduced the levels of alpha-amino adipic acid, a biomarker of cancer, in circulation to levels observed in tumor-free mice. Additionally, alpha-amino adipic acid was reduced in xenografts by ketogenic diets. Moreover, the ketogenic diets increased sphingomyelin levels in plasma and the hydroxylation of sphingomyelins and acylcarnitines in tumors.

**Conclusions:**

Ketogenic diets induced antitumor effects toward melanoma regardless of the tumors´ genetic background, its metabolic signature, and the host immune status. Moreover, ketogenic diets simultaneously affected multiple metabolic pathways to create an unfavorable environment for melanoma cell proliferation, supporting their potential as a complementary nutritional approach to melanoma therapy.

**Supplementary Information:**

The online version contains supplementary material available at 10.1186/s40170-022-00288-7.

## Background

Dietary intervention to exploit the metabolic vulnerabilities of cancers has become a highly attractive approach to target tumor cells [1]. For instance, low-carbohydrate/high-fat ketogenic diets (KDs) have been proposed to target altered glucose metabolism in cancers by reducing the levels of circulating glucose and insulin and insulin-like growth factor-1 to perturb activation of oncogenic signaling cascades downstream of the insulin receptor [2, 3]. The efficacy of KDs, especially their ability to sensitize cancer cells to therapeutic drugs or radiotherapy, has been demonstrated in several preclinical studies [2]. KDs enhance the efficacy of phosphatidylinositol-3 kinase (PI3K) inhibitors and overcome drug resistance in a number of mouse cancer models [3]. However, evidence supporting the efficacy of KDs in melanoma is sparse and conflicting. In one study, KDs accelerated tumor growth in a BRAF mutant melanoma mouse model, whereas the growth of NRAS mutant and BRAF wild-type melanomas remained unaffected, suggesting that the tumor genetic background might determine KD responsiveness [4]. In contrast, a KD successfully enhanced immune checkpoint therapy in an immunocompetent transgenic melanoma mouse model [5]. Moreover, another study reported reduced tumor growth of BRAF-inhibitor-sensitive as well as BRAF-inhibitor-resistant human melanoma xenografts induced by a KD [6]. Since cutaneous melanoma is the major cause of skin-cancer-related death and one of the most frequently metastasizing and drug-resistant types of solid tumor [7], it is essential to clarify the efficacy of KDs as an adjunct cancer therapy.

The oncogenic driver mutations BRAF and NRAS, found in approximately 50% and 30% of melanomas, respectively, contribute to constitutive activation of mitogen activated protein kinase (MAPK) and PI3K pathway signaling, resulting in unabated cell growth, but rendering melanomas susceptible to inhibition of BRAF or its downstream target MEK [8, 9]. However, resistance to BRAF/MEK inhibitors is a major impediment in melanoma therapy [10], in which melanoma cells acquire resistance due to their impressive metabolic flexibility, ranging from glycolytic over glutaminolytic to lipogenic/lipolytic phenotypes that altogether, are influenced by both intrinsic oncogenic activation and extrinsic microenvironmental factors [11, 12]. Melanoma cells mainly utilize glycolysis to produce ATP; however, they are also able to generate energy via mitochondrial respiration [13–16]. Using metabolic profiling, we recently showed that different human melanoma cells engrafted into mice presented distinct metabolomes that was otherwise independent of the intrinsic driver mutation [17].

As a therapeutic strategy in melanoma, KDs offer the potential to ‘rewire’ multiple metabolic pathways simultaneously. In addition to affecting glucose metabolism, KDs were reported to induce redox stress, alter amino acid metabolism and interfere with oncogenic signaling via the production of ketone bodies [2]. Thus, we investigated the effect of KDs on genetically and metabolically heterogeneous human melanoma cells engrafted into mice. For the first time, we have observed reductions in tumor growth regardless of the xenograft characteristics. Moreover, we demonstrate that KDs reduce metastasis in a syngeneic melanoma mouse model. This broad antitumor effect prompted us to look for shared metabolic alterations as possible mediators of the antiproliferative effect of the KDs. We identify distinct alterations in amino acid and lipid metabolism induced by the KDs.

## Methods

### Cells lines

A375 (BRAF^V600E^/NRAS^wt^) cells were purchased from Sigma-Aldrich. WM47 (BRAF^V600E^/NRAS^wt^) and WM3311 (BRAF/NRAS/NF1 wild-type) were provided by Meenhard Herlyn (Wistar Institute, Philadelphia, USA). WM3000 (BRAF^wt^/NRAS^Q61R^) cells were obtained from Rockland. Super-metastatic B16-M4b cells were produced by several rounds of in vivo selection from B16F10 melanoma cells (ATCC) as previously described [18]. A375, WM47, WM3311, and WM3000 cells were cultured as described previously [14]. B16-M4b cells were cultured in DMEM GLUTAMAX high glucose (61965026, Thermo Fisher) supplemented with 10% FBS (F7524, Sigma-Aldrich). All cells were tested as mycoplasma free and kept at 37 °C in a 5% CO2 atmosphere.

### Proliferation assay

Human melanoma cells were seeded in 96-well plates (2 × 10^3^ cells/well for A375 cells and 4 × 10^3^ cells/well for WM47, WM3311 and WM3000 cells). After 24 h, the cultures were supplemented with 2.5 mM, 5 mM, and 10 mM beta-hydroxybutyrate (BHB) (Sigma Aldrich), lithium acetoacetic acid (LiAcAc) (Sigma-Aldrich), or lithium chloride (LiCl) (Alfa Aesar) dissolved freshly prior to the assay in Tu10% medium containing 5 mM glucose. As a vehicle control, cells were kept with cell culture medium alone. Cell viability was measured by crystal violet staining as performed previously [19].

### Diet composition and energy content

Mice were equally assigned to the control (CTRL) diet, long-chain triglyceride-based KD (LCT), and long-chain triglyceride-based KD supplemented with C8 and C10 medium-chain triglycerides (LCT-MCT) (Ssniff-Spezialdiäten). All diets were given ad libitum. Diets were fortified with equal amounts of vitamins and mineral supplements. Detailed information on the diet composition and energy content is provided in Table S1.

### Melanoma xenografts

Mouse xenograft studies were performed in accordance with the Salzburg Animal Care and Use Committee (Study approval no. 20901-TVG/122/6-2018). Animals were maintained under specific pathogen-free conditions and care conformed to the Austria Act on Animal Experimentation. Mice had ad libitum access to water and chow. For the establishment of melanoma xenografts, the human melanoma cell lines A375, WM47, WM3311 and WM3000 were used. 1 × 10^7^ cells in 200 μl of a 1:1 mixture of Matrigel (Corning) and serum-free medium were subcutaneously injected into the right flank of 5- to 7-week-old female CD-1 nude mice (Charles River). Tumor volume was measured every 3–4 days using a caliper and calculated according to the formula: width × height × length/2. In addition, body weight was recorded. Once the tumor volume reached 100 mm^3^, mice were equally assigned to CTRL, LCT or LCT-MCT diets (Table S1) (*n* = 10–13 per group). Blood glucose and BHB levels were monitored at least once per week using the enzyme-based Precision Xceed System (Abbott). WM47 and WM3311 melanoma-bearing mice were euthanized once the tumor volume reached ~ 1000 mm^3^. A375 and WM3000 melanoma-bearing mice were euthanized once the tumor volume reached 10% of net body weight (2000–2500 mm^3^). Mice which approached > 20% net body weight loss were euthanized immediately regardless of tumor size. Blood was taken by cardiac puncture, collected in Lithium Heparin tubes and centrifuged for 3 min at 2000×g. Plasma samples were stored at − 80 °C until metabolomics analysis. Tumors were harvested and divided in two parts of which one half was snap frozen in liquid nitrogen and stored at – 80 °C for metabolomics analysis while the other half was formalin-fixed and paraffin embedded for histological analysis.

### B16-M4b allografts

B16-M4b allograft studies were conducted under approval of the Université catholique de Louvain (UCLouvain) authorities (Comité d’Ethique Facultaire pour l’Expérimentation Animale) according to national animal care regulations (Study approval no. 2016/UCL/MD/018). B16-M4b melanoma allografts were established as described previously [18]. In brief, 100 μl of a 10% Matrigel (Corning)/90% serum free medium cell suspension containing 1 × 10^6^ cells were subcutaneously injected into the right flank of 8-week-old male C57BL/6j mice (Janvier) (day 0). The size of the primary tumor was measured using an electronic caliper, and the tumor volume was calculated using the formula of a prolate ellipsoid. Once the primary tumor reached a diameter of ~ 10 mm (day 11 ± 1), the tumor was surgically removed. One day after the surgery, mice were equally assigned to the CTRL, LCT or LCT-MCT diet groups (Table S1) (*n* = 8–9 per group). Tumor recurrence was monitored after surgery. The size of secondary tumors was measured as described above. In addition, body weight was monitored over time. After 8 days of dietary intervention, blood glucose and BHB levels were measured using the enzyme-based Precision Xceed System (Abbott). Once the secondary tumor of one mouse reached a diameter of ~ 17 mm (day 25 ± 1), all mice were terminally euthanized. Afterwards, lung metastases were counted under a stereoscopic microscope.

### Haematoxylin and eosin staining

Formalin-fixed and paraffin embedded (4 μm) sections of the xenografts were stained with haematoxylin and eosin (HE). In brief, after deparaffination, rehydrated tissue sections were incubated for 6 min in Mayer’s haemalum solution (Merck). Counterstaining was performed using a 0.25% Eosin Y (Merck) solution in 70% ethanol. Slides were mounted with Histokitt (Karl Hecht). The percentage of the necrotic area in the tumor sections was scored from 0 to 100% by a pathologist.

### Immunohistochemistry

Immunohistochemical staining was performed as described previously [20] using 4-μm deparaffinized tumor sections of A375, WM47, WM3311, and WM3000 xenografts from mice fed a CTRL diet to evaluate differences in mitochondrial respiratory complexes and glycolytic markers. The following antibodies and dilutions were used: anti-NDUFS4 (1:1000, ab137064 Abcam), anti-SDHA (1:2000, ab14715 Abcam), anti-MTCO1 (1:1000, ab14705 Abcam), anti-HK2 (1:200, 2867 Cell Signaling), and anti-GLUT1 (1:600, 21829-1-AP Proteintech). For detection, an EnVision kit (Dako) was used according to the manufacturer’s instructions. Scoring of immunohistochemical staining was performed by a pathologist as described previously [13]. For CI, CII, CIV, and HK2 (mitochondrial and cytoplasmic proteins) staining, the staining intensity was scored on a scale of 0–3 (0: no staining; 1: weak staining; 2: moderate staining; 3: strong staining) and multiplied by the percentage of cells stained with the respective intensity to obtain overall score values for statistical analysis as follows: score value = [(0 × Percentage intensity 0) + (1 × Percentage intensity 1) + (2 × Percentage intensity 2) + (3 × Percentage intensity 3)]/100. For GLUT1 (membrane protein) staining, the percentage of positive cells was scored from 0 to 100%.

### Metabolomics

Targeted metabolic profiling of mouse plasma and xenograft samples was performed using the MxP^®^ Quant 500 kit combined with the UHPLC-MS/MS-based acylcarnitine assay (Biocrates Life Sciences) as described previously [17]. In brief, xenograft tissues were homogenized in 85:15 ethanol:0.01 M phosphate lysis buffer at 4 °C. The homogenates were centrifuged at 10,000×g for 2 min at 2–4 °C, supernatants were collected and stored at – 80 °C until analysis.

For the MxP^®^ Quant 500 kit, plasma samples and tissue homogenate supernatants were derivatized using a 5% phenyl-isothiocyanate solution prior to metabolite extraction using 5 mM ammonium acetate in methanol. Extracts were analyzed using a Waters ACQUITY UPLC coupled with a Waters TQ-S MS, using electrospray ionization and multiple reaction mode. Amino acids, biogenic amines, and other small molecules were analyzed by liquid chromatography in 2 different injections and lipids were analyzed by flow injection.

For the acylcarnitine assay, acylcarnitines were extracted using methanol and extracts were analyzed using a Dionex UltiMate 3000 UPLC system coupled to a TSQ^TM^ Vantage MS. All measurements were carried out in positive ion multiple reaction mode.

Data of the MxP^®^ Quant 500 assay were exported and quantified using MetIDQ^TM^ software (Biocrates Life Sciences). Raw data files of the acylcarnitine assay were generated by using Xcalibur^TM^ software (Thermo Fisher) and exported to MetIDQ^TM^ for further analysis, including peak integration, retention time correction, area calculation, calibration curve preparation, and concentration calculation.

Data for tumor tissue were normalized using the tissue factor in MetIDQ^TM^. Quality control samples-based data normalization was performed to minimize the variation of analyses. Initial data cleaning was performed by excluding metabolites with > 20% missing values or values below the limit of detection (LOD) in all experimental groups. Thus, all metabolites with > 80% of the concentration values above the LOD in at least one of the experimental groups were included for statistical analysis. Remaining missing values were replaced by 1/5 of the minimum positive value of each variable once data were analyzed using the web-based tool MetaboAnalyst 5.0 (https://www.metaboanalyst.ca).

### Data analysis

Univariate and multivariate analyses of normalized and log-transformed metabolomics data were carried out using MetaboAnalyst 5.0 (https://www.metaboanalyst.ca) [21]. Statistical analyses of all other data, including metabolic indicators, were performed using GraphPad Prism 9. Group variations are indicated using SD. Group differences were considered significant at *p* ≤ 0.05 and a trend at 0.05 < *p* ≤ 0.10.

For metabolomics data, significant changes in metabolite levels were identified by one-way ANOVA followed by Fisher´s LSD post hoc test. To minimize false positives, false discovery rates (FDRs) were calculated based on the Benjamini-Hochberg procedure [22]. FDR-corrected *p* values < 0.05 were considered statistically significant. Unsupervised principal component analysis (PCA) was performed whenever necessary to determine group separation. To increase the robustness of our analyses, we filtered plasma as well as tumor metabolites and lipids based on FDR-corrected *p* values < 0.05 for CTRL vs. LCT and CTRL vs. LCT-MCT and focused on those metabolites that were significantly altered by both KDs in all four melanoma models (A375, WM47, WM3311, and WM3000). For metabolic pathway analysis (MetPA) of differential metabolites, we only considered metabolites that were significantly altered by both KDs and grouped LCT and LCT-MCT samples together. The Mus musculus and Homo sapiens KEGG (Kyoto Encyclopedia of Genes and Genomes) pathway library was used as a reference for MetPA of mouse plasma metabolites and human melanoma cell xenograft tissue metabolites, respectively. Heatmaps were created using MetaboAnalyst 5.0, and Venn diagrams were generated by using the http://bioinformatics.psb.ugent.be/webtools/Venn website. To identify metabolites (biomarkers) which are increased in melanoma-bearing mice compared to non-tumor mice (NTM) and normalized by KDs, correlation analysis using the Pattern Search tool of MetaboAnalyst was performed [23]. The predefined pattern “1–2–1–1” used for the Pattern Search corresponds to concentrations of metabolites of the different groups in the following order: “NTM CTRL (1)–melanoma CTRL (2)–melanoma LCT (1)–melanoma LCT-MCT (1)”, while “2” indicates higher metabolite concentrations than “1”. The metabolomics data set of plasma samples from NTM was reported previously [17].

## Results

### Ketogenic diets slow the growth of melanoma xenografts independently of oncogenic driver and metabolic signature

**Fig. 1:**
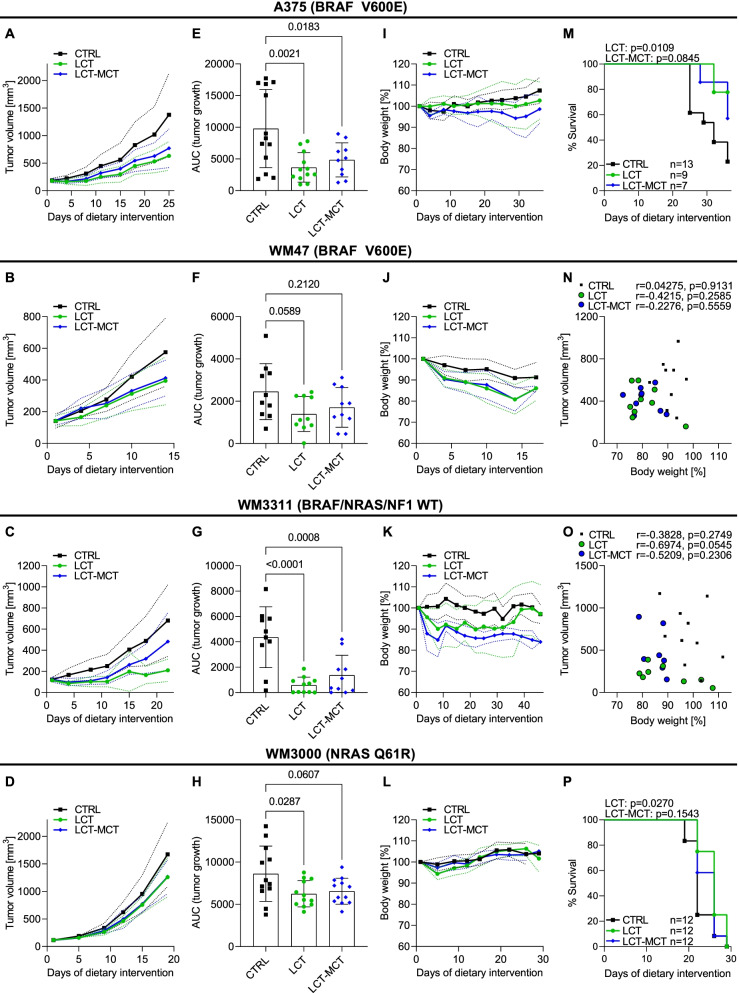
Ketogenic diets slow the growth of melanoma xenografts independently of oncogenic driver and metabolic signature. **A**–**D** Growth curves **A** A375, **B** WM47, **C** WM3311, and **D** WM3000 melanoma xenografts treated with CTRL, LCT, or LCT-MCT diet. Mean tumor volume ± SD is shown for each group until the first CTRL mouse tumor reached the termination size; *n* = 10–13. See also Fig. S2. **E**–**H**. The area under the growth curve (AUC) was calculated for every mouse. AUCs are shown as individual data points ± SD; *p* values were determined by a one-way ANOVA with Dunnett's multiple comparisons test. **I**–**L**. Body weight of **I** A375, **J** WM47, **K** WM3311, and **L** WM3000 melanoma bearing mice during dietary intervention. Net body weight is shown as % of the initial body weight. Individual data points ± SD; *n* = 10–13. See also Fig. S4. **M**, **P**. Survival of **M** A375 and **P** WM3000 melanoma-bearing mice treated with CTRL, LCT or LCT-MCT (since weight loss > 20% led to the elimination of the respective animals, survival analysis based on tumor growth was not possible for the WM47 and WM3311 xenografts). LCT and LCT-MCT survival curves were compared with CTRL and *p* values were determined by a Log-rank (Mantel-Cox) test. **N**, **O**. Correlation analysis between tumor volume and % of initial body weight on day 14 and 22 of **N** WM47 and **O** WM3311 melanoma-bearing mice. Pearson correlation was used to compute Pearson *r* coefficients and the respective *p* values for CTRL, LCT and LCT-MCT groups separately (*n* = 9–10 for CTRL, *n* = 8–9 for LCT and *n* = 7–9 for LCT-MCT groups)

To examine the effect of KDs on melanoma growth, we established BRAF mutant (A375 and WM47), BRAF/NRAS/NF1 wild-type (WM3311), and NRAS mutant (WM3000) xenografts in CD-1 nude mice. As previously reported, A375, WM47, WM3311, and WM3000 cells differ in their glycolytic and respiratory activities [14] and have distinct metabolic profiles [17]. Moreover, the expression levels of mitochondrial oxidative phosphorylation (OXPHOS) complexes CI, CII, and CIV, glucose transporter 1 (GLUT1) and hexokinase 2 (HK2) varied substantially across xenografts (Fig. S1). Since the fat content of KDs can influence their antitumor efficacy [24], we investigated the effect of two KDs (LCT and LCT-MCT, ratio of fat to the sum of carbohydrate and protein of 8:1, Table S1) on melanoma growth. Compared to the CTRL diet, the LCT-based KD slowed the growth of A375, WM3311, and WM3000 xenografts (Fig. 1A, C–E, G, 1H and Fig. S2), and also tended to reduce the growth rate of WM47 xenografts (0.05 < *p* ≤ 0.10) (Fig. 1B, F and Fig. S2). The LCT-MCT-based KD slowed the growth of A375 and WM3311 xenografts and tended to lower the growth rate of WM3000 xenografts (Fig. 1A, C–E, G, H and Fig. S2). Both KDs increased the level of necrosis in A375 and WM3000 tumors compared to CTRL (Fig. S3). Overall, the KDs exerted antiproliferative effects irrespective of the underlying melanoma driver mutations and metabolic plasticity. The influence of the KDs on body weight was quite heterogeneous among melanoma-bearing mice (Fig. 1I–L and Fig. S4). For instance, the body weight of A375- and WM3000-bearing mice was stable or increased in all groups throughout the dietary intervention, whereas WM47- and WM3311-bearing mice lost weight over time, and the weight loss was more pronounced in the KD groups. However, we observed no significant correlation between tumor volume and body weight of WM47- and WM3311-bearing mice (Fig. 1N, O). Thus, the observed antitumor effects of the KDs occurred independently of body weight changes. Since weight reduction > 20% led to the removal of the respective animals, survival analysis based on tumor growth was not possible for the WM47 and WM3311 xenografts. Nevertheless, the LCT-based KD prolonged the survival of weight-stable A375- and WM3000-bearing mice (Fig. 1M, P).

### The level of diet-induced ketosis does not correlate with antitumor effects

**Fig. 2:**
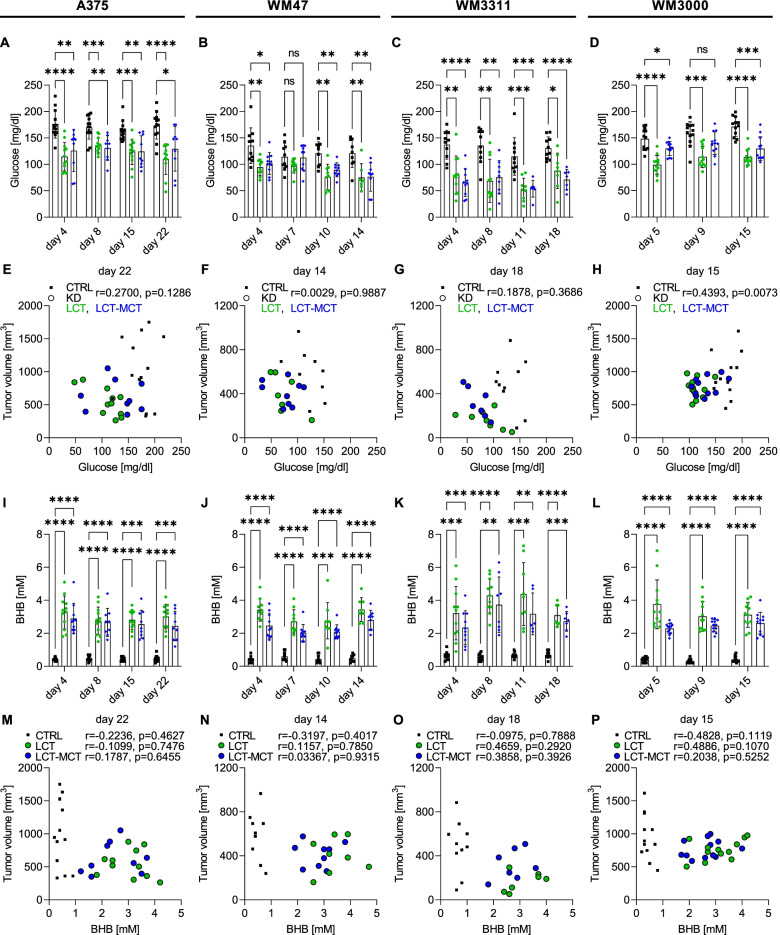
The level of diet-induced ketosis does not correlate with antitumor effects. **A**–**D** Blood glucose and **I**–**L** blood beta-hydroxybutyrate (BHB) concentrations of **A**, **I** A375, **B**, **J** WM47, **C**, **K** WM3311, and **D**, **L** WM3000 melanoma-bearing mice treated with CTRL, LCT, or LCT-MCT diet at different time points throughout the dietary intervention. Individual data points ± SD; *n* = 10–13; *p* values were determined by a mixed-effects analysis or two-way ANOVA with Dunnett's multiple comparisons test; *p < 0.05, **p < 0.01, ***p < 0.001, ****p < 0.0001. **E**–**H** Correlation analysis between tumor volume and glucose concentration of **E** A375, **F** WM47, **G** WM3311, and **H** WM3000 melanoma-bearing mice on the day of the last glucose measurement. Pearson correlation was used to compute Pearson *r* coefficients and respective *p* values for CTRL and KD groups combined (*n* = 33 for A375, *n* = 25 for WM47, *n* = 25 for WM3311, *n* = 36 for WM3000). **M**–**P** Correlation analysis between tumor volume and BHB concentration of **M** A375, **N** WM47, **O** WM3311, and **P** WM3000 melanoma-bearing mice on the day of the last BHB measurement. Pearson correlation was used to compute Pearson *r* coefficients and respective *p* values for CTRL, LCT and LCT-MCT groups separately (*n* = 9–13 for CTRL, *n* = 7–12 for LCT and *n* = 7–12 for LCT-MCT groups)

The antitumor potential of KDs has been associated to their glucose- as well as insulin-lowering effects [2, 25, 26]. Both KDs reduced blood glucose levels by an average of 31% (range 24–48%) (Fig. 2A–D). However, a significant correlation between plasma glucose level and xenograft size was observed only for WM3000-bearing mice (Fig. 2E–H). The ketone body BHB was increased by both KDs by an average of 525% (range 441–651%) in melanoma-bearing mice (Fig. 2I–L). Whereas MCT-supplementation of KDs has been reported to enhance ketone body levels in humans [27], we did not observe this effect in the present study. Furthermore, we did not find a negative correlation between plasma BHB level and xenograft size in KD-fed mice (Fig. 2M–P). Treatment of A375, WM47, WM3311, and WM3000 cells with 2.5, 5, and 10 mM BHB in vitro did not impact their proliferation (Fig. S5). Acetoacetic acid (AcAc), the metabolic precursor of BHB was recently reported to confer tumor-accelerating effects of a KD in BRAF mutant melanoma-bearing mice [4]. We also treated the melanoma cell lines with the lithium salt form of AcAc (LiAcAc), which is more stable than the acid form, as well as the control salt (LiCl) in vitro. Both LiAcAc and LiCl reduced the proliferation of the melanoma cell lines, but only at high concentrations, indicating that Li^+^ ions and not AcAc are responsible for the growth-inhibitory effects of LiAcAc, as previously observed for other cancer cells [19].

### Ketogenic diets reduce plasma levels of essential amino acids and the cancer biomarker alpha-amino adipic acid

**Fig. 3:**
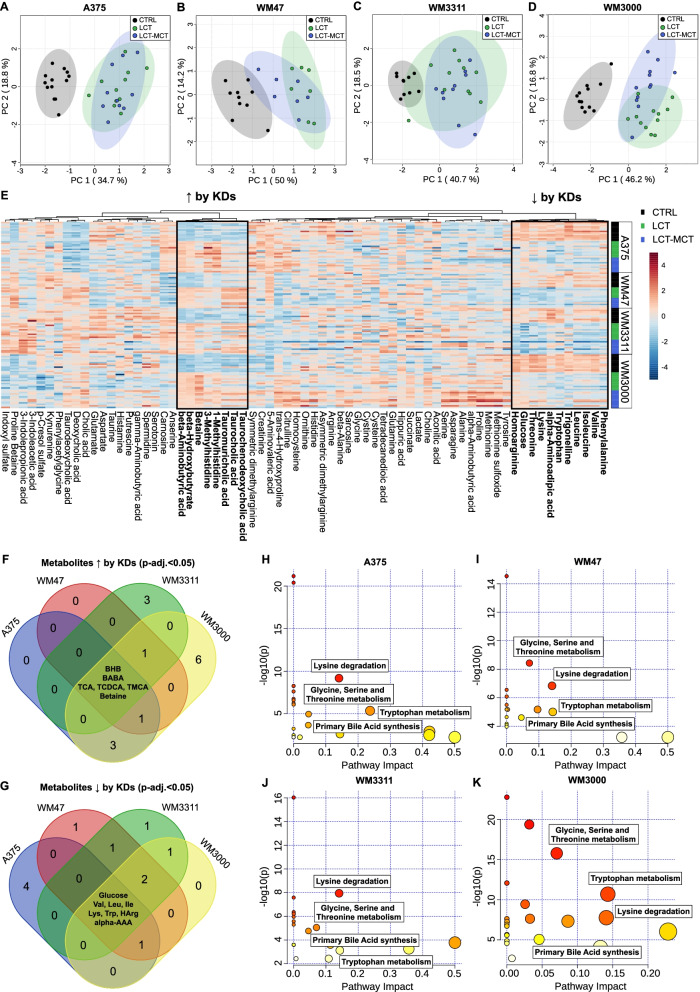
Ketogenic diets induce metabolic changes in plasma of melanoma xenograft-bearing mice beyond glucose reduction and ketosis. **A**–**D** Principal component analysis (PCA) of polar metabolite profiling data obtained from plasma samples of **A** A375, **B** WM47, **C** WM3311, and **D** WM3000 xenograft-bearing mice treated with CTRL, LCT, or LCT-MCT diet. *n* = 7–13. **E** Heatmap of metabolites quantified in plasma of xenograft-bearing mice. The two clusters highlighted in the heatmap represent up- or downregulated metabolites by KDs vs. CTRL. *n* = 7–13. **F**–**G** Identification of significant differential metabolites in plasma between both KDs and CTRL for each xenograft model by one-way ANOVA and Fisher´s LSD post hoc test. Plasma metabolites were filtered based on an FDR-adjusted *p* value < 0.05 for both KDs vs. CTRL. The Venn diagrams represent the intersections among the 4 xenograft models (A375, WM47, WM3311, WM3000) for significantly **F** upregulated and **G** downregulated plasma metabolites (see also Table S4). **H**–**K** Overview of metabolic pathway analysis (MetPA) using plasma metabolites of **H** A375, **I** WM47, **J** WM3311, and **K** WM3000 melanoma-bearing mice. Pathways consistently altered by KDs across the melanoma models are highlighted in the MetPA results overview, indicating matched pathways arranged by *p* values from pathway enrichment analysis (*Y*-axis) and pathway impact values from pathway topology analysis (*X*-axis). Node color and radius are based on the *p* value and pathway impact value, respectively. *n* = 10–13 in CTRL groups and *n* = 14-24 in KD groups. Alpha-AAA: alpha-aminoadipic acid, BABA: beta-aminobutyric acid, BHB: beta-hydroxybutyrate, HArg, homoarginine, Ile: isoleucine, Leu: leucine, Lys: lysine, TCA: taurocholic acid, TCDCA: taurochenodeoxycholic acid, TMCA: Tauromuricholic acid, Trp: tryptophan, Val: valine, ↑: increased, ↓: decreased

To gain a better understanding of which metabolic changes are elicited by KDs in melanoma-bearing mice beyond simply just the reduction of glucose and the increase in ketone bodies, we profiled the plasma metabolome using targeted metabolomics. We quantified 67 polar metabolites and added mean glucose and BHB concentrations to the metabolomics data analysis (Table S2). For each xenograft model, PCA revealed a clear separation of plasma samples obtained from CTRL- and KD-fed animals (Fig. 3A–D). Hierarchical clustering analysis uncovered two distinct clusters of KD regulation: up- and downregulated metabolites (Fig. 3E). We found 25, 27, 31, and 48 polar metabolites to be significantly different in the plasma of A375-, WM47-, WM3311-, and WM3000-bearing mice, respectively, between at least 2 dietary intervention groups (Table S3). Filtering of the metabolites based on a FDR-adjusted *p* value < 0.05 for both CTRL vs. LCT and CTRL vs. LCT-MCT revealed that the KDs consistently upregulated BHB, beta-aminobutyric acid, 3 taurine-conjugated bile acids, and betaine (Fig. 3F, Table S4). Moreover, glucose, the essential amino acids (EAAs) lysine, tryptophan, valine, leucine, and isoleucine, as well as alpha-amino adipic acid and homoarginine were consistently downregulated (Fig. 3G, Table S4). MetPA revealed that the KDs consistently altered primary bile acid synthesis, glycine-, serine-, and threonine metabolism, lysine degradation, and tryptophan metabolism in A375-, WM47-, WM3311-, and WM3000-bearing mice (with *p* < 0.05 and impact value > 0) (Fig. 3H–K and Table S5).

**Fig. 4:**
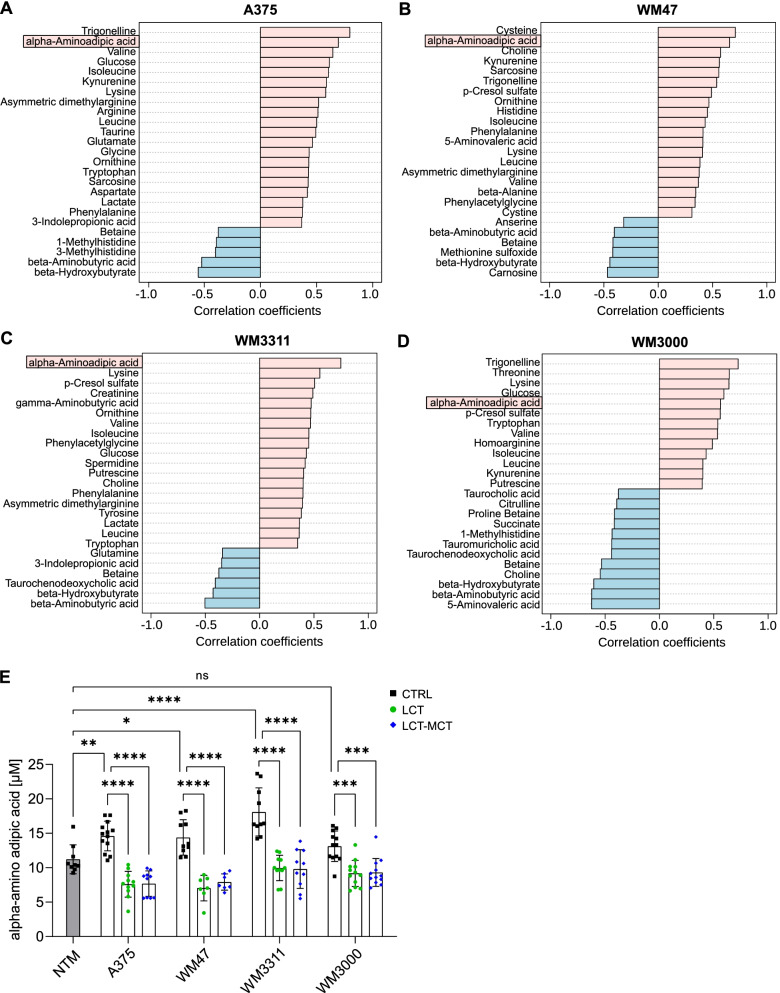
Ketogenic diets normalize the concentration of alpha-amino adipic in melanoma-bearing mice to levels found in non-tumor mice (NTM). **A**–**D** Correlation plots showing the top 25 metabolites that are significantly associated with the predefined pattern “1–2–1–1” in **A** A375, **B** WM47, **C** WM3311, and **D** WM3000 melanoma-bearing mice. The pattern “1–2–1–1” corresponds to concentrations of metabolites of the different groups in the following order: “NTM CTRL (1)–melanoma CTRL (2)–melanoma LCT (1)–melanoma LCT-MCT (1)”, while “2” indicates higher metabolite concentrations than “1”. **E** Absolute μM concentration of alpha-amino adipic acid in plasma of NTM fed with CTRL diet, melanoma-bearing mice fed with CTRL diet and melanoma-bearing mice fed with LCT or LCT-MCT. Individual data points ± SD; *n* = 7–13; *p* values were determined by a one-way ANOVA with Šidák’s multiple comparisons test, *p < 0.05, **p < 0.01, ***p < 0.001, ****p < 0.0001

Using correlation analysis, we identified alpha-amino adipic acid as the only metabolite that significantly correlates with the predefined pattern “1–2–1–1” with a Pearson correlation coefficient *r* > 0.5 in all four melanoma models (Fig. 4A–D and Table S6). This pattern corresponds to lower metabolite concentrations in plasma of NTM fed the CTRL diet and in plasma of melanoma-bearing mice fed with KDs compared to melanoma-bearing mice fed with CTRL diet, respectively. Thus, the KDs normalized the increased levels of alpha-amino adipic acid in melanoma-bearing mice to concentrations found in NTM (Fig. 4E).

### Plasma metabolic alterations induced by ketogenic diets are reflected in the tumor metabolome

As a consequence of metabolic changes induced by KDs, tumors will experience increased or reduced nutrient availability. To evaluate how KDs influence the tumor metabolome, we also profiled polar metabolites in melanoma xenografts.

**Fig. 5:**
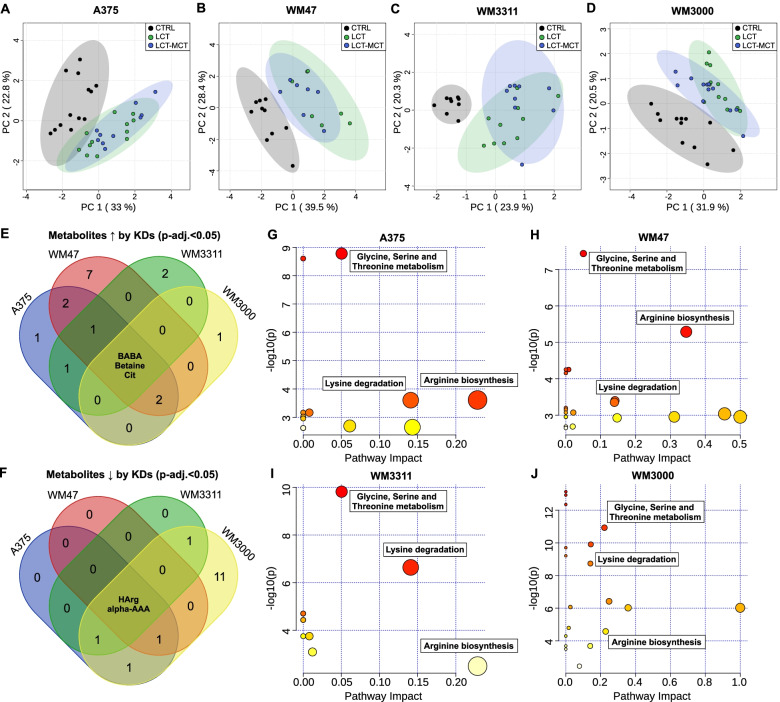
Plasma metabolic alterations induced by ketogenic diets are reflected in the tumor metabolome. **A**–**D** Principal component analysis (PCA) of polar metabolite profiling data obtained from **A** A375, **B** WM47, **C** WM331, and **D** WM3000 melanoma xenografts treated with CTRL, LCT, or LCT-MCT diet. *n* = 7–13. **E**, **F** Identification of significant differential metabolites in tumors between both KDs and CTRL for each xenograft model by one-way ANOVA and Fisher’s LSD post hoc test. Tumor metabolites were filtered based on an FDR-adjusted *p* value < 0.05 for both KDs vs. CTRL. The Venn diagrams represent the intersections among the 4 xenograft models (A375, WM47, WM3311, WM3000) for significantly **E** upregulated and **F** downregulated tumor metabolites (see also Table S9). **G**–**J** Overview of metabolic pathway analysis (MetPA) using tumor metabolites of **G** A375, **H** WM47, **I** WM3311, and **J** WM3000 melanoma-bearing mice. Pathways consistently altered by KDs throughout the melanoma models are highlighted in the MetPA results overview, indicating matched pathways arranged by *p* values from pathway enrichment analysis (*Y*-axis) and pathway impact values from pathway topology analysis (*X*-axis). Node color and radius are based on the *p* value and pathway impact value, respectively. *n* = 9–13 in CTRL groups and *n* = 14–24 in KD groups. Alpha-AAA: alpha-aminoadipic acid, BABA: beta-aminobutyric acid, Cit: Citrulline, HArg, homoarginine, ↑: increased, ↓: decreased

In xenografts, we were able to quantify 62 polar metabolites (Table S7), of which 18, 21, 16, and 26 were found to be significantly different in at least 2 dietary intervention groups in the A375, WM47, WM3311, and WM3000 xenografts, respectively (Table S8). Similar to the observed metabolome differentiation in plasma, PCA displayed a clear separation of tumor samples from CTRL- and KD-fed mice (Fig. 5A–D). To evaluate metabolite changes in tumors subjected to KDs, the same filtering approach was applied as for plasma samples (Fig. 5E, F and Table S9). The KDs induced consistently increased levels of beta-aminobutyric acid, betaine and citrulline in the xenografts (Fig. 5E). Furthermore, the KDs lowered the concentrations of alpha-amino adipic acid and homoarginine in tumors (Fig. 5F). MetPA of significantly different metabolite concentrations in tumors revealed that the KDs consistently altered glycine-, serine-, and threonine metabolism, lysine degradation and arginine biosynthesis (with *p* < 0.05 and impact value > 0) (Fig. 5G–J and Table S10).

Thus, the enhancing effect of KDs on beta-aminobutyric acid and betaine levels and their suppressing effect on alpha-amino adipic acid and homoarginine levels observed in the plasma were reflected in the xenografts. Alterations in glycine-, serine-, and threonine metabolism and lysine degradation were also detected in both, plasma and tumors. Two metabolic alterations induced by KDs in tumors but not in the plasma were upregulation of citrulline levels and arginine biosynthesis.

### Ketogenic diets increase circulating sphingomyelin levels

**Fig. 6:**
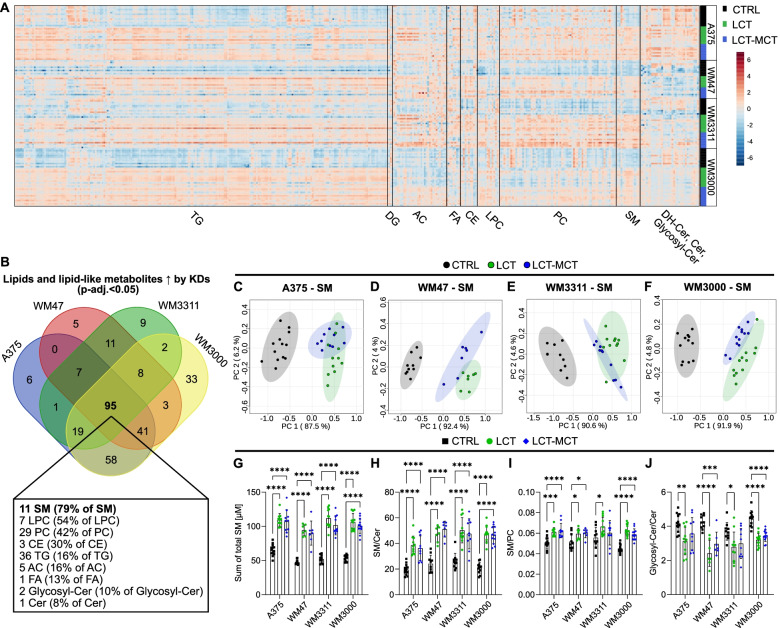
Ketogenic diets alter sphingomyelin levels in plasma of melanoma xenograft-bearing mice. **A** Heatmap representing all lipids and lipid-like metabolites quantified in plasma of xenograft-bearing mice treated with CTRL, LCT, or LCT-MCT diet. Lipids and lipid-like metabolites were grouped by compound class. *n* = 7–13. **B** Identification of significant differential lipids and lipid-like metabolites in plasma between both KDs and CTRL for each xenograft model by one-way ANOVA and Fisher´s LSD post hoc test. Plasma lipids and lipid-like metabolites were filtered based on an FDR-adjusted *p* value < 0.05 for both KDs vs. CTRL. The Venn diagram represents the intersections among the 4 xenograft models (A375, WM47, WM3311, WM3000) for significantly upregulated lipids and lipid-like metabolites in plasma (see also Table S13). Ninety-five lipids and lipid-like metabolites were consistently upregulated by KDs in melanoma xenografts. The box below the Venn diagram indicates how many lipids and lipid-like metabolites per compound class were included in those 95. The percentage indicates the amount of respective lipids and lipid-like metabolites relative to the total number of quantified lipids and lipid-like metabolites per compound class. **C**–**F** Principal component analysis (PCA) of sphingomyelins quantified in plasma samples of **C** A375, **D** WM47, **E** WM3311, and **F** WM3000 xenograft-bearing mice treated with CTRL, LCT, or LCT-MCT. *n* = 7–13. **G**–**J** Effect of KDs on the total sphingomyelin pool in plasma and on metabolic indicators for sphingomyelin synthesis. Sum concentration of **G** total sphingomyelins (hydroxylated and non-hydroxylated sphingomyelins), **H** ratio of sphingomyelins to ceramides, **I** ratio of sphingomyelins to phosphatidylcholines, and **J** ratio of glycosyl-ceramides to ceramides quantified in plasma melanoma-bearing mice treated with CTRL, LCT, or LCT-MCT. Individual data points ± SD; *n* = 7–13; *p* values were determined by a one-way ANOVA with Dunnett’s multiple comparisons test comparing CTRL with each KD group for every xenograft model, *p < 0.05, **p < 0.01, ***p < 0.001, ****p < 0.0001. AC: acylcarnitine, CE: cholesteryl ester, Cer: ceramide, DG: diglyceride, DH-Cer: dihydroceramide, FA: fatty acid, Glycosyl-Cer: glycosylceramide, LPC: lyso-phosphatidylcholine, PC: phosphatidylcholine, SM: sphingomyelin, TG: triglyceride, ↑: increased

We next analyzed the impact of KDs on plasma lipids. In the plasma of melanoma-bearing mice, 404 lipids and lipid-like metabolites were quantified (Fig. 6A and Table S11). Significant differences were found in 294, 268, 192, and 350 lipids and lipid-like metabolites in the plasma of A375-, WM47-, WM3311-, and WM3000-bearing mice, respectively, between at least 2 dietary groups (Table S12). 95 lipids and lipid-like metabolites were consistently upregulated by both KDs in all melanoma xenografts, including 11 sphingomyelins, 7 lyso-phosphatidylcholines, 29, phosphatidylcholines, 3 cholesteryl esters, 36 triglycerides, 5 acylcarnitines, 1 fatty acid, 2 glycosylceramides, and 1 ceramide (Fig. 6B and Table S13). Moreover, the lyso-phosphatidylcholine LPC a C16:1, the cholesteryl ester CE(16:1), and the hexosylceramide HexCer(d18:1/24:1) were consistently downregulated by the KDs (Table S13).

The 11 upregulated sphingomyelins represented 79% of the total sphingomyelin pool quantified in the plasma. PCA of the sphingomyelins indicated strong group separation of CTRL- and KD-fed mice in all melanoma models (Fig. 6C–F). Moreover, the sum concentration of sphingomyelins quantified in the plasma was increased by the KDs (Fig. 6G). Sphingomyelins are synthesized from ceramides and phosphatidylcholines via sphingomyelin synthase (SMS) [28]. Thus, the ratios of sphingomyelins to ceramides as well as to phosphatidylcholines could serve as indicators of SMS activity. The ratio of sphingomyelins to ceramides was increased in the melanoma models by both KDs (Fig. 6H), while the ratio of sphingomyelins to phosphatidylcholines was increased by at least one KD (Fig. 6I). Because sphingomyelins are synthesized from ceramides, fewer ceramides might be available for glycosylation [28]. Indeed, the ratio of glycosylceramides to ceramides was decreased by at least one KD in xenograft-bearing mice (Fig. 6J).

### Ketogenic diets increase the levels of hydroxylated sphingomyelins and acylcarnitines in melanoma xenografts

**Fig. 7:**
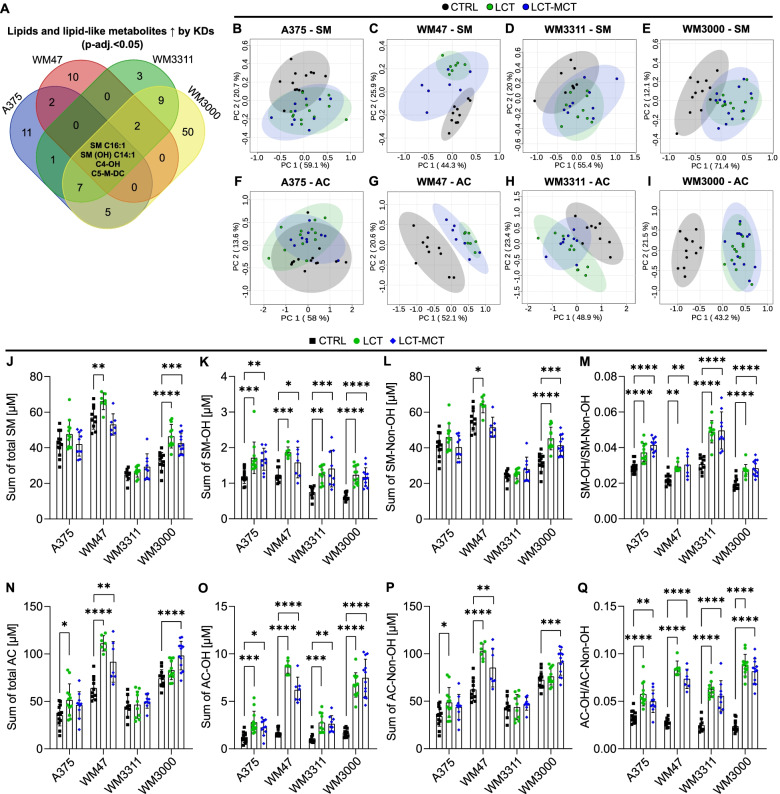
Ketogenic diets increase the levels of hydroxylated sphingomyelins and acylcarnitines in melanoma xenografts. **A** Identification of significant differential lipids and lipid-like metabolites in tumors obtained from melanoma-bearing mice treated with CTRL, LCT, or LCT-MCT diet between both KDs and CTRL for each xenograft model by one-way ANOVA and Fisher´s LSD post hoc test. Tumor lipids and lipid-like metabolites were filtered based on an FDR-adjusted *p* value < 0.05 for both KDs vs. CTRL. The Venn diagram represents the intersections among the 4 xenograft models (A375, WM47, WM3311, WM3000) for significantly upregulated lipids and lipid-like metabolites in tumors (see also Table S16). **B**–**I** Principal component analysis (PCA) of **B**–**E** sphingomyelins and **F**-**I** acylcarnitines quantified in tumor samples of **B**/**F** A375, **C**/**G** WM47, **D**/**H** WM3311, and **E**/**I** WM3000 xenograft-bearing mice treated with CTRL, LCT, or LCT-MCT. *n* = 7–13. **J**–**Q** Effect of KDs on sphingomyelin and acylcarnitine pools in tumor tissues. Sum concentration of total **J** sphingomyelins and **N** acylcarnitines, **K** hydroxylated sphingomyelins and **O** hydroxylated acylcarnitines, **L** non-hydroxylated sphingomyelins and **P** non-hydroxylated acylcarnitines, and ratio of hydroxylated to non-hydroxylated fraction of **M** sphingomyelins and **Q** acylcarnitines quantified in melanoma xenografts treated with CTRL, LCT, or LCT-MCT. Individual data points ± SD; *n* = 7–13; *p* values were determined by a one-way ANOVA with Dunnett’s multiple comparisons test comparing CTRL with each KD group for every xenograft model, *p < 0.05, **p < 0.01, ***p < 0.001, ****p < 0.0001. AC: acylcarnitine, -Non-OH: non-hydroxylated, -OH: hydroxylated, SM: sphingomyelin, ↑: increased

Like for plasma, we also assessed whether KDs affect lipids and lipid-like metabolites in engrafted melanoma tumors. In total, 217 lipids and lipid-like metabolites were quantified in tumors (Table S14). Of these 217 species, the levels of 46, 60, 41, and 142 were significantly different in at least 2 dietary groups in A375, WM47, WM3311, and WM3000 melanomas, respectively (Table S15). Similar to what we observed in plasma, the concentrations of 2 sphingomyelins (SM C16:1 and SM(OH) C14:1) were increased by both KDs in melanoma xenografts (Fig. 7A). Moreover, the levels of 2 acylcarnitines, hydroxybutyrylcarnitine (C4-OH), and methylglutarylcarnitine (C5-M-DC), were increased by KDs in melanoma xenografts (Fig. 7A and Table S16). These sphingomyelins and acylcarnitines were also more abundant in plasma (Table S13). No lipid or lipid-like metabolite was consistently decreased by both KDs in the xenografts (Table S16). PCA separated CTRL and KD-treated tumor samples for sphingomyelin and acylcarnitine data (Fig. 7B–I). However, the sum concentration of sphingomyelins was less affected in tumors compared to the plasma (Fig. 7J). Dividing the total sphingomyelin pool into its hydroxylated and non-hydroxylated fractions revealed that KDs had a strong enhancing effect on the level of hydroxylated sphingomyelins in melanoma xenografts (Fig. 7K, L). Moreover, the ratio of hydroxylated to non-hydroxylated sphingomyelins was increased by both KDs (Fig. 7M). Furthermore, we observed the same effect on hydroxylation for acylcarnitines (Fig. 7N–Q).

### Ketogenic diets reduce metastatic dissemination in the lungs of B16-M4b allograft mice

**Fig. 8:**
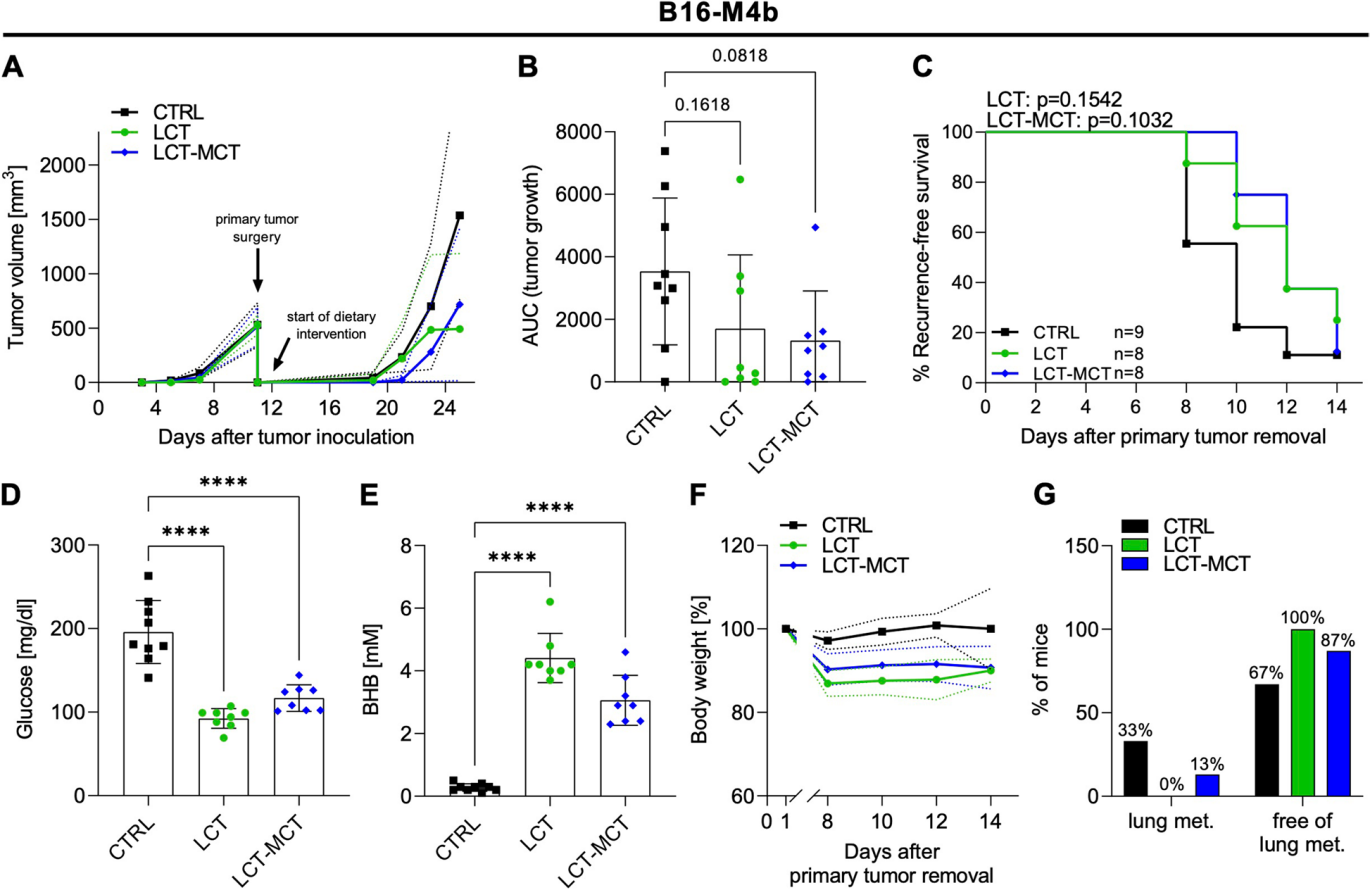
Ketogenic diets reduce metastatic dissemination in the lungs of B16-M4b melanoma allograft mice. **A** Primary growth of B16-M4b allografts in syngeneic C57BL/6j mice, followed by secondary tumor growth after primary tumor removal (day 11 ± 1; tumor diameter of ~ 1 cm). After surgery, mice were equally assigned to CTRL, LCT or LCT-MCT diet. Mean tumor volume ± SD; *n* = 8–9. See also Fig. S6. **B** The area under the growth curve (AUC) was calculated for every mouse. AUCs are shown as individual data points ± SD; *p* values were determined by a one-way ANOVA with Dunnett’s multiple comparisons test. **C** Recurrence-free survival of mice fed CTRL, LCT, or LCT-MCT diet. LCT and LCT-MCT survival curves were compared with CTRL, and *p* values were determined by a log-rank (Mantel-Cox) test. **D** Blood glucose and **E** blood beta-hydroxybutyrate (BHB) concentrations of B16-M4b melanoma-bearing mice treated with CTRL, LCT or LCT-MCT on day 8 of dietary intervention. Individual data points ± SD; *n* = 8–9; *p* values were determined by a one-way ANOVA with Dunnett's multiple comparisons test, ****p < 0.0001. **F** Body weight of B16-M4b melanoma-bearing mice during dietary intervention. Net body weight is shown as % of the initial body weight. Individual data points ± SD; *n* = 8–9. See also Fig. S6. **G** Percentage of mice presenting metastasis or no metastasis in the lungs of B16-M4b melanoma bearing mice after 14 days of dietary intervention

The melanoma xenograft models require the use of immunodeficient mice. Therefore, we also examined the effect of KDs in an immunocompetent mouse model, measuring tumor recurrence after initial surgical therapy. Since the prognosis for patients with metastatic melanoma is poor, we also analyzed the effect of KD intervention on melanoma metastasis. B16-M4b-bearing syngeneic mice were fed with CTRL diet or KDs after removal of the primary tumor. In this aggressive melanoma model, we observed no significant effect of the KDs on tumor recurrence compared to CTRL (Fig. 8A–C and Fig. S6A–S6C). However, recurring tumors in the LCT-MCT-based KD group tended to be smaller after 14 days of dietary intervention compared to those in the CTRL group (Fig. 8B). Both KDs reduced glucose and increased BHB levels in the blood of B16-M4b-bearing mice (Fig. 8D, E). Body weight of mice in the LCT- and LCT-MCT-based KD groups decreased by 10-15% during the first week of the KD intervention and stabilized thereafter (Fig. 8F and Fig. S6D–S6F). Importantly, melanoma lung metastases were not detected in LCT-fed mice and were seen in only 13% of LCT-MCT-fed mice, whereas 33% of CTRL mice developed lung metastases (Fig. 8G).

## Discussion

Metabolic reprogramming in melanoma cells is strongly linked to the constitutive activation of MAPK and PI3K signaling induced by driver mutations such as BRAF and NRAS mutants, leading to various metabolic phenotypes [9, 11, 12, 17]. The four melanoma xenografts used in the present study vary in their metabolome [17] and in their expression levels of respiratory and glycolytic markers. However, despite this genetic and phenotypic variation, our present findings suggest that KDs could be an effective antitumor therapy against melanomas generally.

In contrast to our data, Xia et al. reported a growth-accelerating effect of a KD on BRAF mutant xenografts (including A375), whereas NRAS and wild-type melanomas were unaffected [4]. The authors attributed the tumor-promoting effect to the ketone body AcAc, which was increased by the KD and enhanced the binding of mutant BRAF (but not wild-type BRAF) to MEK1 to promote MEK-ERK signaling and tumor growth [4]. Two major differences distinguish our work from that of Xia et al. First, the authors in that study observed a significant increase in AcAc but not BHB in KD-fed mice (ketogenic ratio 6:1) [4]. Since BHB is the most abundant ketone body in the blood during ketosis, it seems their xenograft-bearing mice did not reach a high level of diet-induced ketosis. In our study, the level of plasma BHB increased by an average of 6.2-fold in KD-fed melanoma-bearing mice (ketogenic ratio 8:1). In previous studies, CD-1 nude mice fed a KD with a ketogenic ratio of 2:1 also exhibited increased BHB [29, 30]. Thus, the 6:1 ketogenic ratio used by Xia and colleagues does not explain the lack of BHB elevation. Second, our study design aimed to mimic a therapeutic setting to test the effect of KDs on melanoma growth in vivo. We started the KD intervention once the xenografts had reached a measurable size, approximately 1 week after tumor cell injection. In contrast, Xia et al. randomized mice to dietary intervention groups 1 week before tumor initiation [4]. Consistent with our data, de Groot et al. demonstrated reduced tumor growth of BRAF-inhibitor-sensitive and BRAF-inhibitor-resistant A375 melanoma xenografts in mice fed with KD [6]. Interestingly, the authors used exactly the same KD as Xia et al. but started KD intervention after tumor cell injection as we did. Thus, the timing of KD administration may significantly influence the response, which needs to be addressed in further studies [31].

Ferrere et al. showed that plasma BHB levels negatively correlated with the size of transgenically induced melanomas in mice treated with a KD (ketogenic ratio 4:1) [5]. The authors confirmed the mode of action by replacing the KD with BHB administration, which slowed tumor growth to the same extent as the KD. Consequently, BHB was concluded to be the bioactive metabolite of the KD, exerting its antiproliferative effect by influencing the tumor-associated T cell activity [5]. In contrast, we observed no correlation between plasma BHB levels of melanoma-bearing mice and tumor size. Our data suggest that neither BHB nor AcAc has a direct effect on melanoma cell proliferation. Moreover, in our xenograft model, the antiproliferative effects of KDs cannot be attributed to a T cell response because the CD-1 immune-compromised mice that we used lack T cells. Treatment of immunocompetent syngeneic B16-M4b-bearing mice with KDs after primary tumor removal only tended to reduce recurrent tumor growth, even though plasma BHB levels were elevated. Thus, a BHB-mediated immune response does not explain how KDs reduce tumor growth. One reason for the insignificant effect of KDs on the secondary growth of B16-M4b-allografts could be the aggressiveness of the model or because the KDs were applied as a monotherapy, as opposed to an adjunct therapy. However, fewer B16-M4b-bearing KD- than CTRL-fed mice developed lung metastases, indicating that KDs not only affect primary tumor growth but can also reduce metastasis. This is consistent with findings in mice injected with B16 melanoma cells via the tail vein and subsequently fed either sucrose or pure vegetable oil, in which mice provided vegetable oil displayed a ~ 66% reduction in lung metastases [32].

Our KDs reduced the plasma levels of several EAAs, which is in agreement with previous studies showing that KDs have a profound effect on amino acid metabolism [24, 33, 34]. One common feature of cancers, including melanomas, is the upregulation of EAA transporters to meet the high EAA demand necessary for proliferation [35, 36]. The branched-chain amino acids (BCAAs) valine, leucine and isoleucine can serve as alternative carbon fuels for the TCA cycle, and mediate lipogenesis through acetyl-CoA [37, 38]. Elevated levels of circulating BCAAs and increased expression of BCAA-metabolizing enzymes in tumors were found in patients with various cancers, indicating an increased requirement for BCAAs as anaplerotic substrates [39]. Accordingly, leucine deprivation triggers apoptotic death in melanoma cells [40]. Thus, reduced availability of EAAs, including BCAAs in the blood induced by KDs might directly impair tumor survival [41, 42]. One plausible explanation for why KDs reduce EAA levels could be their lower protein content compared to CTRL diets (Table S1). However, in a previous study we found neither a reduction of plasma EAA levels nor a reduction in tumor growth rate in neuroblastoma-bearing mice fed a control diet containing the same low amount and the same source of protein as used in the KD in the present study [24]. Thus, KDs used in the present and previous study have a potent effect on EAA metabolism irrespective of their low protein content.

MetPA revealed that KDs altered lysine degradation both in plasma and in xenografts. Lysine is a pivotal EAA, since lysine-rich proteins include ones indispensable for cellular structure and function (e.g., ribosome assembly) [41]. Since lysine is required to synthesize homoarginine from arginine [43], the reduced availability of lysine could probably explain the reduction in homoarginine levels observed in plasma and tumors. In addition, the level of alpha-amino adipic acid, a product of lysine metabolism, was reduced by the KDs in both plasma and xenografts. Compared to tumor-free mice, melanoma-bearing mice showed increased levels of alpha-amino adipic acid in plasma which got reversed by the KDs. Alpha-amino adipic acid served as a biomarker to distinguish mouse embryonic fibroblasts with and without deficiency in the oncogene KLF4 [44]. Moreover, alpha-amino adipic acid has been reported as a prognostic biomarker for prostate cancer [45]. Furthermore, high levels of alpha-amino adipic acid correlated with poor survival of patients with glioblastoma [46]. However, whether the antiproliferative effects of KDs on melanoma cells relate to reductions in lysine and its related metabolites homoarginine and alpha-amino adipic acid remains to be elucidated.

Numerous alterations of the lipid metabolic network are known to contribute to sustained cell proliferation and melanoma metastasis [47]. We observed major differences among the melanoma xenografts in terms of sphingolipid and glycerophospholipid species, which are bioactive molecules important for plasma membrane homeostasis [17]. Even though melanoma subtypes present heterogeneous lipid profiles, our KDs consistently upregulated the levels of lipids of different classes in the plasma of melanoma-bearing mice. Notably, the plasma concentrations of the majority of sphingomyelins were increased by the KDs. Enriched sphingolipid and glycerophospholipid metabolites were also observed in the plasma of patients who consumed a KD for 2 weeks [48]. SMS, the enzyme which produces sphingomyelins from ceramides and phosphatidylcholines, is often downregulated in melanoma and this is associated with worse outcomes [49]. KDs increased the ratio of plasma sphingomyelins to ceramides as well as to phosphatidylcholines, potentially indicating increased SMS activity. In the xenografts, the ratio of hydroxylated to non-hydroxylated sphingomyelins as well as acylcarnitines was elevated by our KDs. Reduced expression of fatty acid 2-hydroxylase, the enzyme that catalyzes hydroxylation of free fatty acids prior to their incorporation into 2-hydroxylated sphingolipids [50], was detected in tumor tissue from colorectal and gastric cancer patients [51, 52]. Fatty acid 2-hydroxylation suppressed colorectal tumorigenesis and metastasis in vitro and inhibited gastric cancer growth by increasing sensitivity to cisplatin in mice [51, 52]. Thus, the present data suggest that KDs could exert antitumor effects via significant alterations in sphingomyelin metabolism and hydroxylation of certain lipid species.

## Conclusions

Taken together, our data indicate that KDs induce antitumor effects toward melanoma and affect different metabolic pathways concomitantly, creating an unfavorable metabolic environment for cancer cell proliferation. The effects of our KDs on the body weight of melanoma-bearing mice appear to depend more on the cell lines injected than on the diet itself. Several clinical studies reported weight reduction in cancer patients adhering to a KD; however, the KDs were sufficient to preserve lean mass while reducing total fat mass in these patients [53–55]. Importantly, KDs induced weight gain and maintenance of a positive nitrogen balance in cachectic cancer patients [56]. A clear benefit of KDs is their reported potential to sensitize tumor cells to chemo- and radiotherapy [2]. Thus, combining KDs with BRAF/MEK or PI3K inhibitors may synergize with the tumor-reductive effect exerted by KDs as a monotherapy in our study. Compared to anticancer drugs and standard treatments, KDs are inexpensive, fairly easy to implement when monitored appropriately, and well tolerated, which leads us to propose KDs as part of a multimodal therapy to improve the outcomes of classic cancer therapy [57–59]. Clinical trials investigating the effect of KDs in cancer patients are ongoing (https://clinicaltrials.gov), and results from these trials will be essential to assess the efficacy and feasibility of KDs in clinical practice.

## Supplementary Information

**Additional file 1: Fig. S1.** Expression of OXPHOS and glycolysis markers in melanoma xenografts. A-E Immunohistochemical staining of A OXPHOS complex I (CI), B complex II (CII), C complex IV (CIV), D hexokinase 2 (HK2), and E glucose transporter 1 (GLUT1) in A375, WM47, WM3311, and WM3000 tumors from mice fed a control diet. Individual data points and median; n = 5-6; *p* values were determined by a Kruskal-Wallis test with Dunn's multiple comparisons test; **p<0.01, ***p<0.001. Images show representative CI, CII, CIV, HK2, and GLUT1 staining in A375-, WM47-, WM3311-, and WM3000-xenografts. Scale bar = 100 μm. **Fig. S2.** Tumor growth of melanoma xenografts from mice fed a control diet or KDs. A-L Tumor growth curves of A-C A375, D-F WM47, G-I WM3311 and J-L WM3000 melanoma xenografts in single CD-1 nude mice treated with CTRL, LCT or LCT-MCT diet. **Fig. S3.** Effect of KDs on tumor necrosis in melanoma xenografts. A-D Percentage of necrosis in A A375, B WM47, C WM3311, and D WM3000 tumors from mice treated with CTRL, LCT or LCT-MCT diet scored in haematoxylin and eosin (HE) stained histological sections of xenografts. Individual data point and median; n = 6-12; *p* values were determined by a Kruskal-Wallis test with Dunn's multiple comparisons test, *p<0.05, **p<0.01. Images show representative HE stained A375-, WM47-, WM3311-, and WM3000-xenografts from mice fed a CTRL, LCT or LCT-MCT diet. Scale bar = 200 μm. **Fig. S4.** Body weight of melanoma xenograft-bearing mice treated with a control diet or KDs. A-L Body weight curves of A-C A375, D-F WM47, G-I WM3311 and J-L WM3000 melanoma-bearing single CD-1 nude mice treated with CTRL, LCT or LCT-MCT diet. Net body weight is shown as % of the initial body weight. Body weight loss >20% was a termination criterion. **Fig. S5.** Beta-hydroxybutyrate and acetoacetate have no effect on proliferation of human melanoma cells *in vitro*. A-D Treatment of human melanoma cell lines A A375, B WM47, C WM3311, and D WM3000 with 2.5, 5, and 10 mM beta-hydroxybutyrate (BHB), lithium-acetoacetate (LiAcAc) and lithium-chloride (LiCl). *p* values were determines by a one-way ANOVA with Dunnett's multiple comparisons test; n = 8 from 2 independent experiments. **Fig. S6.** Tumor growth and body weight of B16-M4b-melanoma bearing syngeneic C57BL/6j mice treated with a control diet or KDs. A-C Tumor growth curves of single B16-M4b melanoma allografts treated with A CTRL, B LCT or C LCT-MCT diet. D-F Body weight curves of single B16-M4b-melanoma bearing mice treated with D CTRL, E LCT or F LCT-MCT diet. Net body weight is shown as % of the initial body weight. Body weight loss >20% was a termination criterion.

**Additional file 2: Table S1.** Composition of control and ketogenic diets. **Table S2.** Metabolites quantified in plasma of melanoma-bearing mice treated with control or ketogenic diets. Related to Fig. 3. **Table S3.** One-way ANOVA results from control vs. ketogenic diet group comparisons of metabolites quantified in plasma of melanoma-bearing mice. Related to Fig. 3. **Table S4.** Overview and filtering result of significantly up- and down-regulated metabolites in plasma of melanoma-bearing mice treated with control and ketogenic diets. Related to Fig. 3. **Table S5.** Metabolic pathway analysis (MetPA) results from control vs. ketogenic diet group comparisons of metabolites quantified in plasma of melanoma-bearing mice. Related to Fig. 3. **Table S6.** Correlation analysis results for plasma metabolites with the pattern 1-2-1-1 in non-tumor mice versus melanoma-bearing mice treated with control or ketogenic diets. Related to Fig. 4. **Table S7.** Polar metabolites quantified in xenograft tissues of melanoma-bearing mice treated with control or ketogenic diets. Related to Fig. 5. **Table S8.** One-way ANOVA results from control vs. ketogenic diet group comparisons of polar metabolites quantified in xenograft tissues of melanoma-bearing mice. Related to Fig. 5. **Table S9.** Overview and filtering result of significantly up- and down-regulated polar metabolites in plasma of melanoma-bearing mice treated with control and ketogenic diets. Related to Fig. 5. **Table S10.** Metabolic pathway analysis (MetPA) results from control vs. ketogenic diet group comparisons of polar metabolites quantified in xenograft tissues of melanoma-bearing mice. Related to Fig. 5. **Table S11.** Lipids and lipid-like metabolites quantified in plasma of melanoma-bearing mice treated with control or ketogenic diets. Related to Fig. 6. **Table S12.** One-way ANOVA results from control vs. ketogenic diet group comparisons of lipids and lipid-like metabolites quantified in plasma of melanoma-bearing mice. Related to Fig. 6. **Table S13.** Overview and filtering result of significantly up- and down-regulated lipids and lipid-like metabolites in plasma of melanoma-bearing mice treated with control and ketogenic diets. Related to Fig. 6. **Table S14.** Lipids and lipid-like metabolites quantified in xenograft tissues of melanoma-bearing mice treated with control or ketogenic diets. Related to Fig. 7. **Table S15.** One-way ANOVA results from control vs. ketogenic diet group comparisons of lipids and lipid-like metabolites quantified in xenograft tissue of melanoma-bearing mice. Related to Fig. 7. **Table S16.** Overview and filtering result of significantly up- and down-regulated lipids and lipid-like metabolites in xenograft tissue of melanoma-bearing mice treated with control and ketogenic diets. Related to Fig. 7.

## Data Availability

All data that support the findings of this study are included in the article and its supporting information. Metabolomics data of samples from melanoma-bearing mice treated with CTRL diet and KDs is available at the NIH Common Fund’s National Metabolomics Data Repository (NMDR) website, the Metabolomics Workbench, https://www.metabolomicsworkbench.org where it has been assigned Project ID PR001198. The data can be accessed directly via it's Project DOI: 10.21228/M8VD7F. This work is supported by NIH grant U2C-DK119886. Metabolomics data of samples from non-tumor mice was published recently [17] and is available at 10.5281/zenodo.4457420.

## Abbreviations

AcAc: Acetoacetic acid
BABA: Beta-aminobutyric acid
BCAAs: Branched-chain amino acids
BHB: Beta-hydroxybutyrate
CTRL: Control
EAAs: Essential amino acids
FDR: False discovery rate
GLUT1: Glucose transporter 1
HK2: Hexokinase 2
KD: Ketogenic diet
KEGG: Kyoto Encyclopedia of Genes and Genomes
LCT: Long-chain triglyceride-based ketogenic diet
LCT-MCT: Long-chain triglyceride-based ketogenic diet supplemented with C8 and C10 medium chain triglycerides
LiAcAc: Lithium acetoacetic acid
LiCl: Lithium chloride
LOD: Limit of detection
MAPK: Mitogen-activated protein kinase
MetPA: Metabolic pathway analysis
MS: Mass spectrometry
NTM: Non-tumor mice
OXPHOS: Oxidative phosphorylation
PCA: Principal component analysis
PI3K: Phosphatidylinositol-3 kinase
SMS: Sphingomyelin synthase
UPLC: Ultra performance liquid chromatography
