# Supplementary material for: Ketogenic diets slow melanoma growth in vivo regardless of tumor genetics and metabolic plasticity
Source: Cancer Metab. 2022 Jul 18;10:12. doi: 10.1186/s40170-022-00288-7 (PMC9290281; doi:10.1186/s40170-022-00288-7)
Supplement: Supplementary file 1 — Additional file 1: Fig. S1. Expression of OXPHOS and glycolysis markers in melanoma xenografts. A-E Immunohistochemical staining of A OXPHOS complex I (CI), B complex II (CII), C complex IV (CIV), D hexokinase 2 (HK2), and E glucose transporter 1 (GLUT1) in A375, WM47, WM3311, and WM3000 tumors from mice fed a control diet. Individual data points and median; n = 5-6; p values were determined by a Kruskal-Wallis test with Dunn's multiple comparisons test; **p<0.01, ***p<0.001. Images show representative CI, CII, CIV, HK2, and GLUT1 staining in A375-, WM47-, WM3311-, and WM3000-xenografts. Scale bar = 100 μm. Fig. S2. Tumor growth of melanoma xenografts from mice fed a control diet or KDs. A-L Tumor growth curves of A-C A375, D-F WM47, G-I WM3311 and J-L WM3000 melanoma xenografts in single CD-1 nude mice treated with CTRL, LCT or LCT-MCT diet. Fig. S3. Effect of KDs on tumor necrosis in melanoma xenografts. A-D Percentage of necrosis in A A375, B WM47, C WM3311, and D WM3000 tumors from mice treated with CTRL, LCT or LCT-MCT diet scored in haematoxylin and eosin (HE) stained histological sections of xenografts. Individual data point and median; n = 6-12; p values were determined by a Kruskal-Wallis test with Dunn's multiple comparisons test, *p<0.05, **p<0.01. Images show representative HE stained A375-, WM47-, WM3311-, and WM3000-xenografts from mice fed a CTRL, LCT or LCT-MCT diet. Scale bar = 200 μm. Fig. S4. Body weight of melanoma xenograft-bearing mice treated with a control diet or KDs. A-L Body weight curves of A-C A375, D-F WM47, G-I WM3311 and J-L WM3000 melanoma-bearing single CD-1 nude mice treated with CTRL, LCT or LCT-MCT diet. Net body weight is shown as % of the initial body weight. Body weight loss >20% was a termination criterion. Fig. S5. Beta-hydroxybutyrate and acetoacetate have no effect on proliferation of human melanoma cells in vitro. A-D Treatment of human melanoma cell lines A A375, B WM47, C WM3311, and D WM3000 with 2.5, 5, and 10 mM beta [file 40170_2022_288_MOESM1_ESM.docx]

**Supplementary Figures**

**Ketogenic diets slow melanoma growth *in vivo* regardless of tumor genetics and metabolic plasticity**

Daniela D Weber, Sepideh Aminzadeh-Gohari, Maheshwor Thapa, Anna-Sophia Redtenbacher, Luca Catalano, Tânia Capelôa, Thibaut Vazeille, Michael Emberger, Thomas K Felder, René G Feichtinger, Peter Koelblinger, Guido Dallmann, Pierre Sonveaux, Roland Lang, and Barbara Kofler


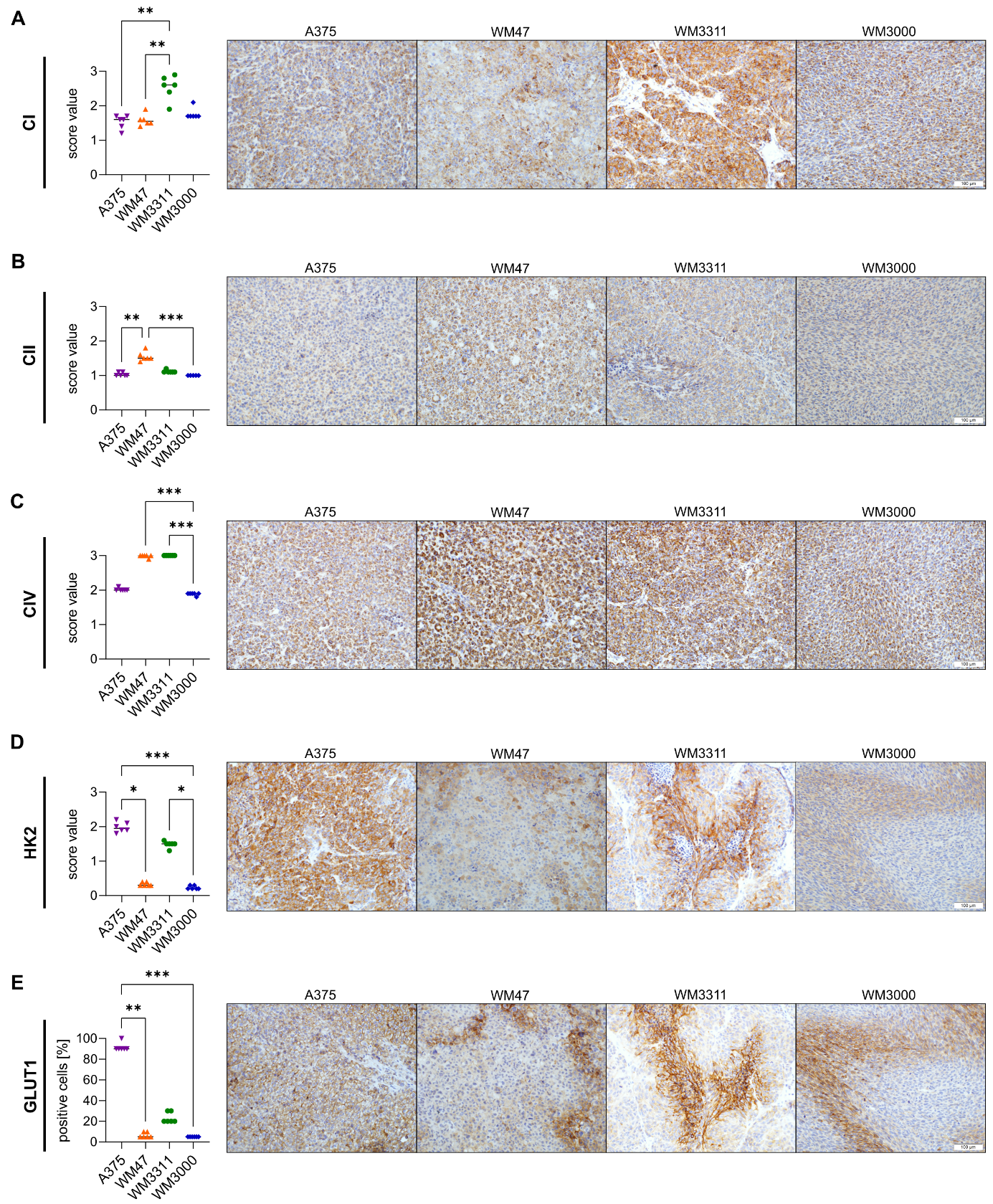


**Fig. S1** Expression of OXPHOS and glycolysis markers in melanoma xenografts. **A**-**E** Immunohistochemical staining of **A** OXPHOS complex I (CI), **B** complex II (CII), **C** complex IV (CIV), **D** hexokinase 2 (HK2), and **E** glucose transporter 1 (GLUT1) in A375, WM47, WM3311, and WM3000 tumors from mice fed a control diet. Individual data points and median; n = 5-6; p values were determined by a Kruskal-Wallis test with Dunn's multiple comparisons test; **p<0.01, ***p<0.001. Images show representative CI, CII, CIV, HK2, and GLUT1 staining in A375-, WM47-, WM3311-, and WM3000-xenografts. Scale bar = 100 µm.


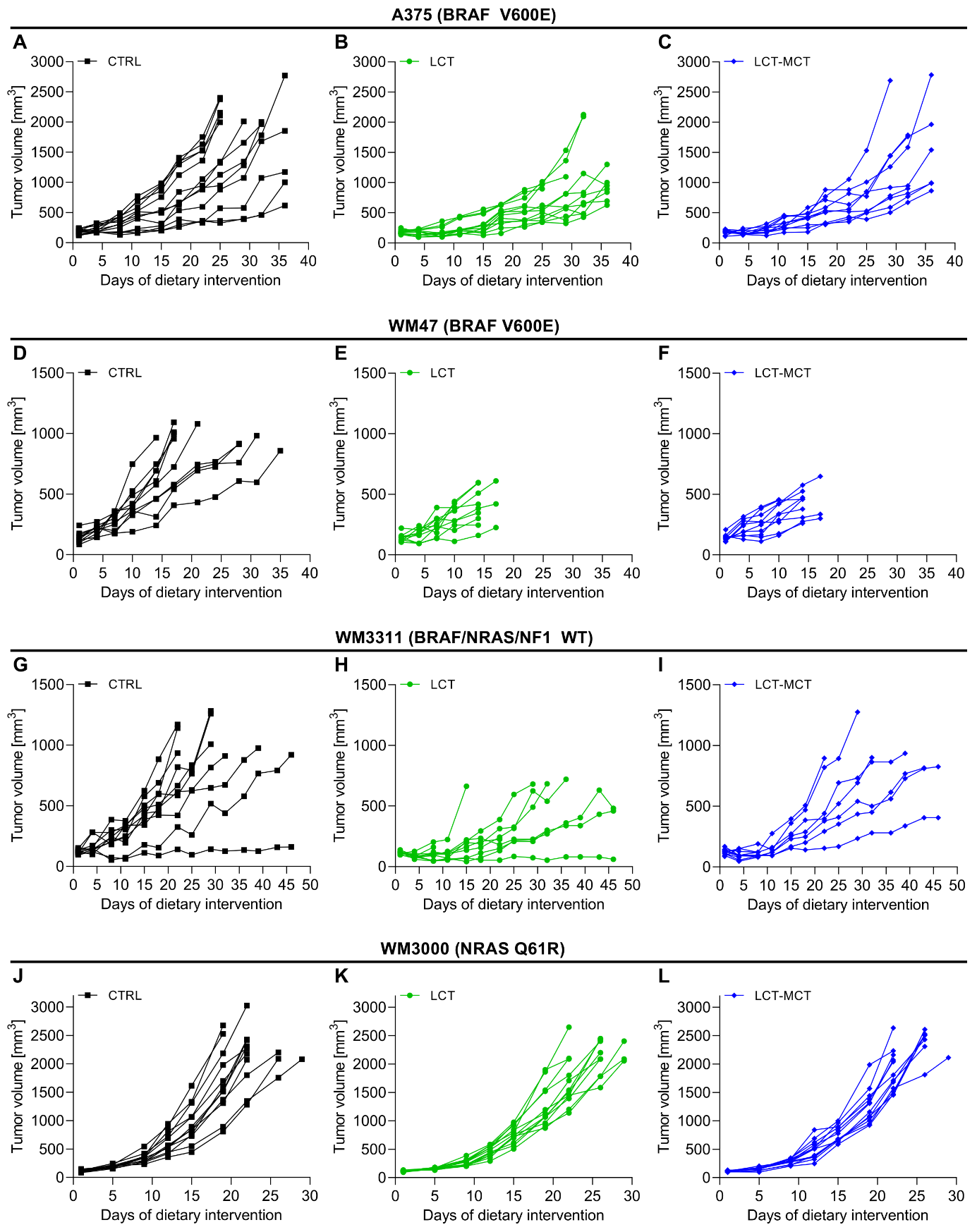


**Fig. S2** Tumor growth of melanoma xenografts from mice fed a control diet or KDs. **A**-**L** Tumor growth curves of **A**-**C** A375, **D**-**F** WM47, **G**-**I** WM3311 and **J**-**L** WM3000 melanoma xenografts in single CD-1 nude mice treated with CTRL, LCT or LCT-MCT diet.


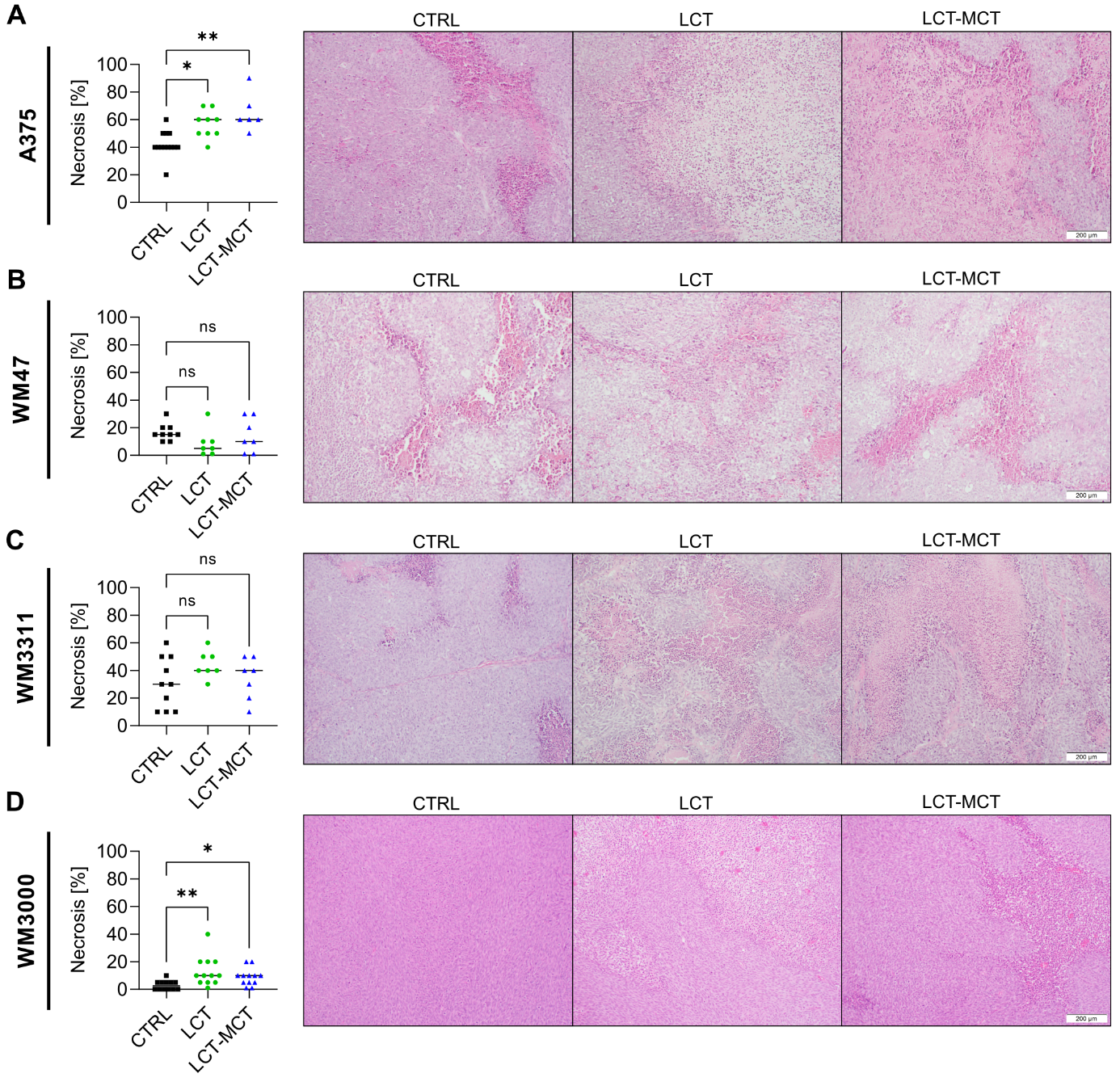


**Fig. S3** Effect of KDs on tumor necrosis in melanoma xenografts. **A**-**D** Percentage of necrosis in **A** A375, **B** WM47, **C** WM3311, and **D** WM3000 tumors from mice treated with CTRL, LCT or LCT-MCT diet scored in haematoxylin and eosin (HE) stained histological sections of xenografts. Individual data point and median; n = 6-12; p values were determined by a Kruskal-Wallis test with Dunn's multiple comparisons test, *p<0.05, **p<0.01. Images show representative HE stained A375-, WM47-, WM3311-, and WM3000-xenografts from mice fed a CTRL, LCT or LCT-MCT diet. Scale bar = 200 µm.


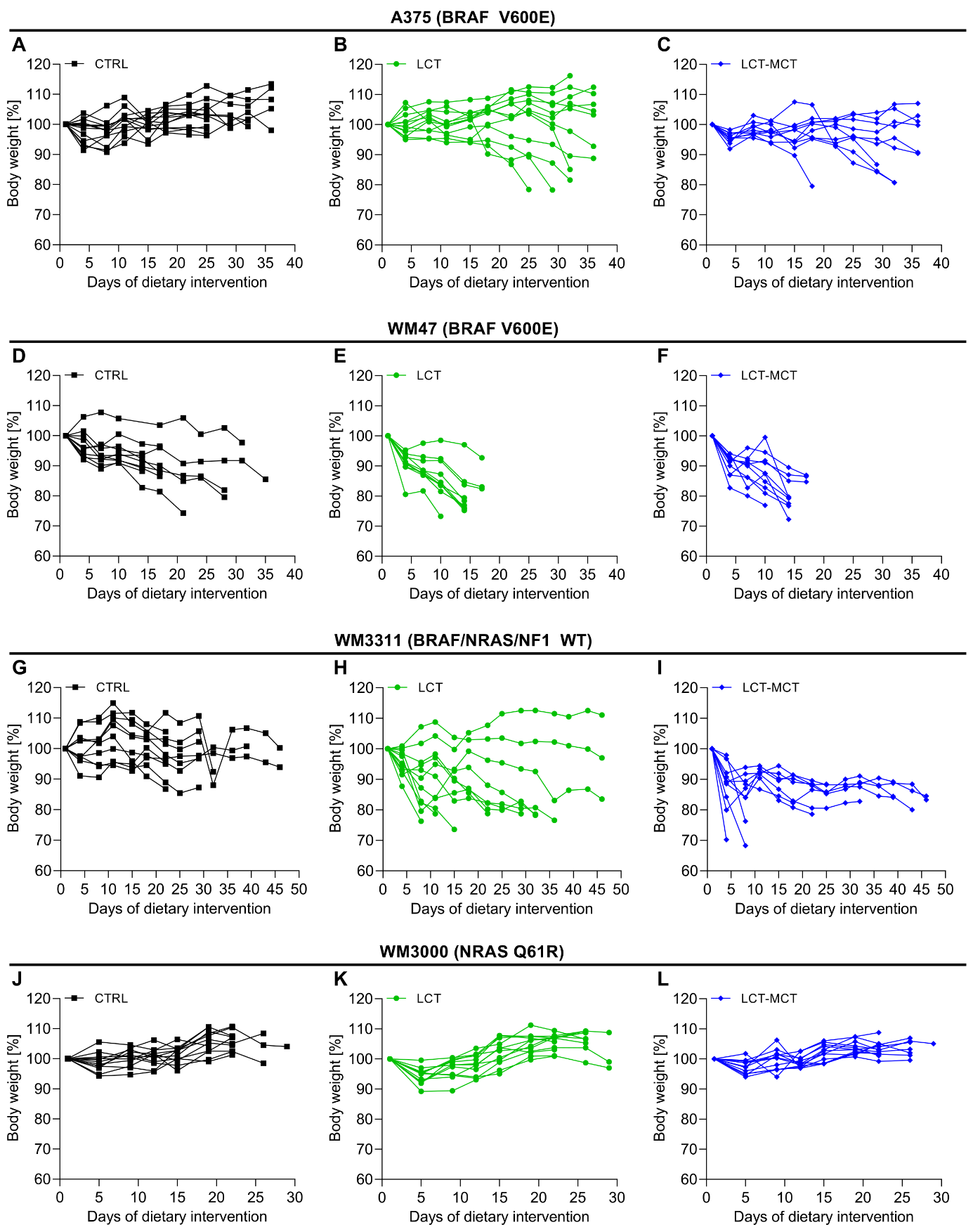


**Fig. S4** Body weight of melanoma xenograft-bearing mice treated with a control diet or KDs. **A**-**L** Body weight curves of **A**-**C** A375, **D**-**F** WM47, **G**-**I** WM3311 and **J**-**L** WM3000 melanoma-bearing single CD-1 nude mice treated with CTRL, LCT or LCT-MCT diet. Net body weight is shown as % of the initial body weight. Body weight loss >20% was a termination criterion.


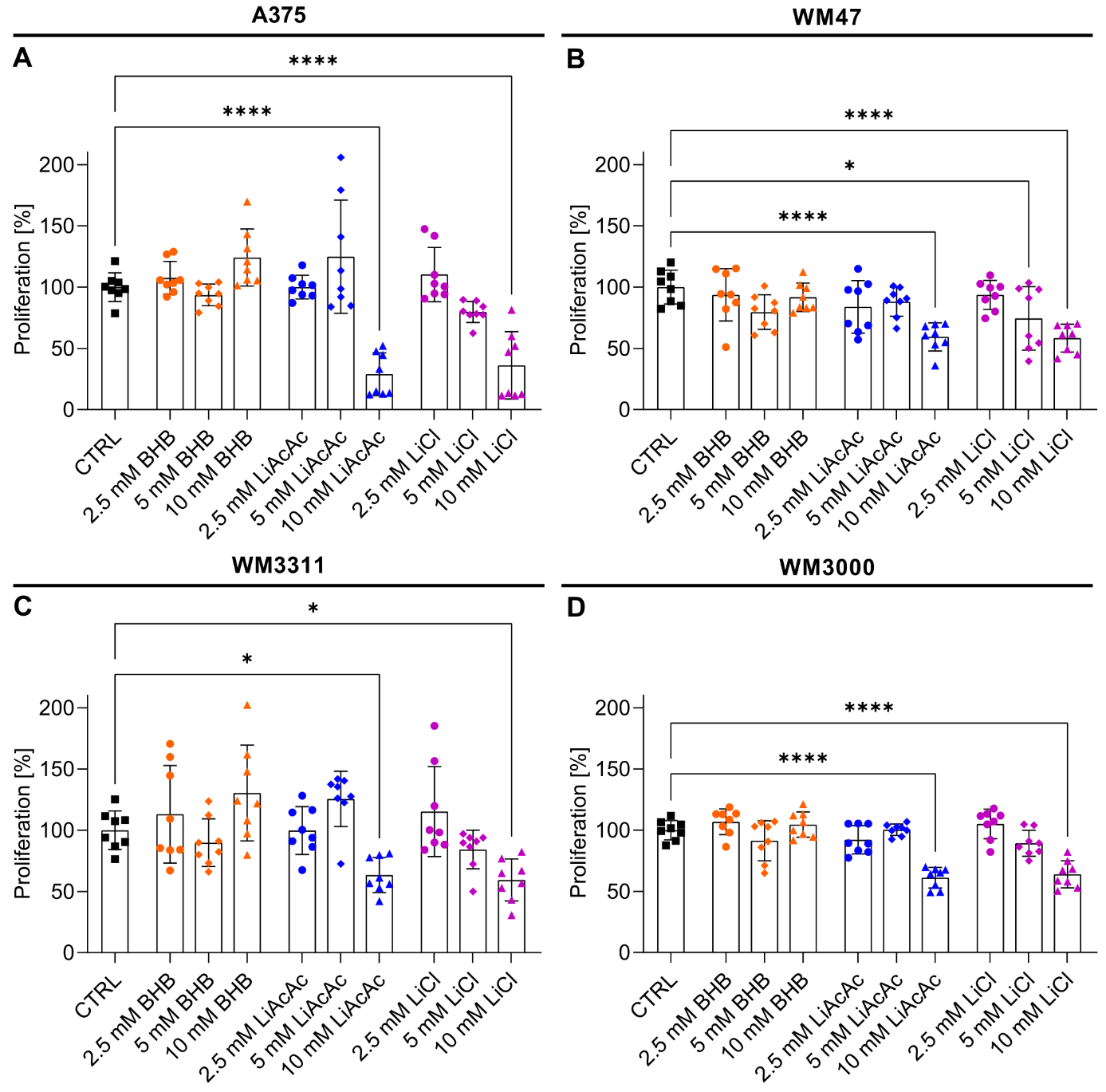


**Fig. S5** Beta-hydroxybutyrate and acetoacetate have no effect on proliferation of human melanoma cells *in vitro*. **A**-**D** Treatment of human melanoma cell lines **A** A375, **B** WM47, **C** WM3311, and **D** WM3000 with 2.5, 5, and 10 mM beta-hydroxybutyrate (BHB), lithium-acetoacetate (LiAcAc) and lithium-chloride (LiCl). p values were determines by a one-way ANOVA with Dunnett's multiple comparisons test; n = 8 from 2 independent experiments.


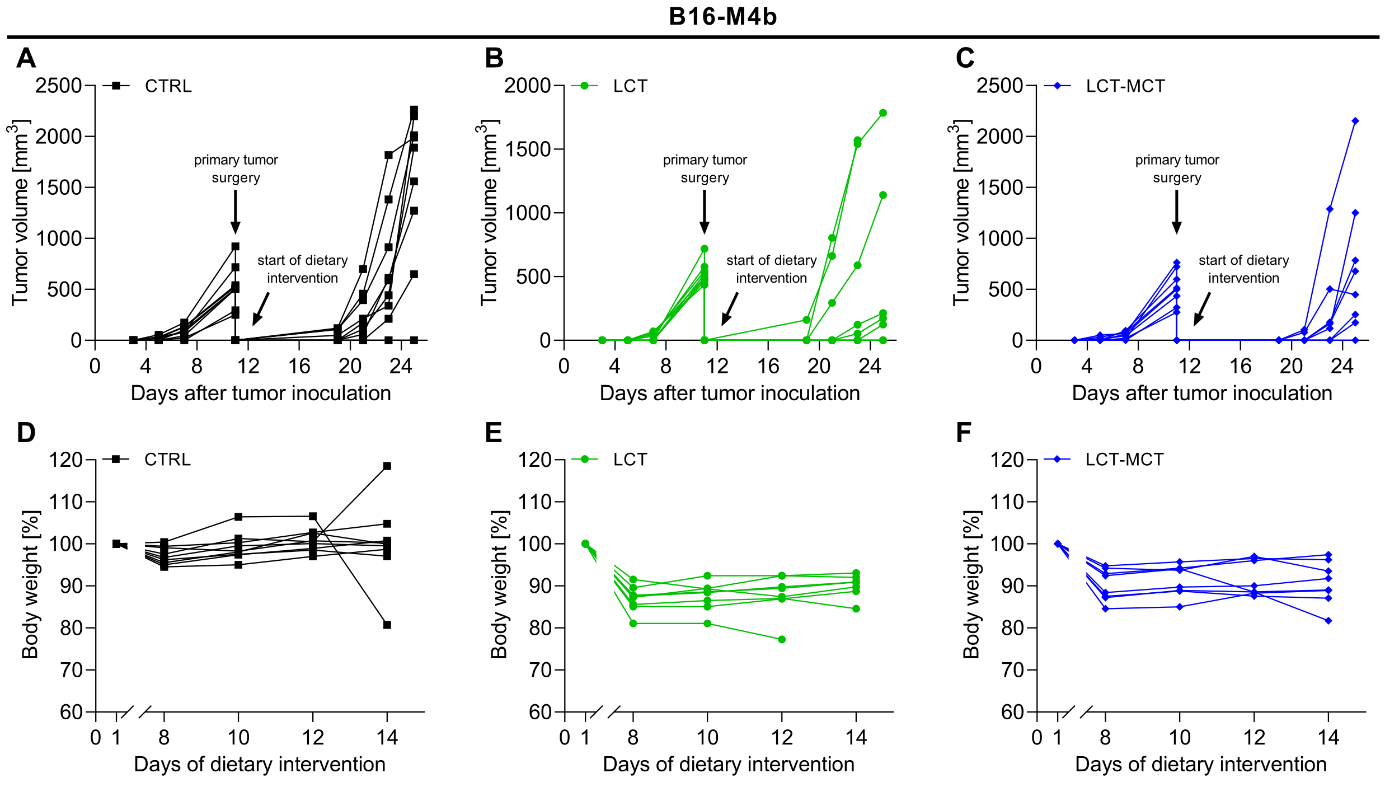


**Fig. S6** Tumor growth and body weight of B16-M4b-melanoma bearing syngeneic C57BL/6j mice treated with a control diet or KDs. **A**-**C** Tumor growth curves of single B16-M4b melanoma allografts treated with **A** CTRL, **B** LCT or **C** LCT-MCT diet. **D**-**F** Body weight curves of single B16-M4b-melanoma bearing mice treated with **D** CTRL, **E** LCT or **F** LCT-MCT diet. Net body weight is shown as % of the initial body weight. Body weight loss >20% was a termination criterion.
